# Supplementary material for: Synthesis and Anti-Trypanosoma cruzi Biological Evaluation of Novel 2-Nitropyrrole Derivatives
Source: Molecules. 2022 Mar 27;27(7):2163. doi: 10.3390/molecules27072163 (PMC9000427; doi:10.3390/molecules27072163)

# Synthesis and anti-*trypanosoma cruzi* biological evaluation of novel 2-nitropyrrole derivatives

**Fanny Mathias [1,2], Youssef Kabri [1], Damien Brun [1], Nicolas Primas [1,3], Carole Di Giorgio [4] and Patrice Vanelle [1,3,*]**

[1] Equipe Pharmaco-Chimie Radicalaire, CNRS, ICR UMR 7273, Faculté de Pharmacie, Aix Marseille University, 27 Boulevard Jean Moulin, CS30064, CEDEX 05, 13385 Marseille, France; fanny.mathias@univ-amu.fr (F.M.); youssef.kabri@univ-amu.fr (Y.K.); brundamien83@gmail.com (D.B.); nicolas.primas@univ-amu.fr (N.P.)
[2] Assistance Publique-Hôpitaux de Marseille (APHM), Pharmacie Usage Intérieur, Hôpital Nord,
Chemin-des-Bourrely, 13015 Marseille, France
[3] Assistance Publique-Hôpitaux de Marseille (APHM), Service Central de la Qualité et de l'Information
Pharmaceutiques (SCQIP), Hôpital de la Conception, 147, boulevard Baille, 13005 Marseille, France
[4] Laboratoire de Mutagénèse Environnementale, CNRS, IRD, Aix Marseille University, IMBE UMR 7263, Avignon University, 13385 Marseille, France; carole.di-giorgio@univ-amu.fr
* Correspondence: patrice.vanelle@univ-amu.fr; Tel.: +33-4-9183-5580

## 3.1 Chemistry

### 3.1.1 Generality

Melting points were determined on a Köfler melting point apparatus (Wagner & Munz GmbH, München, Germany) and were uncorrected. NMR spectra were recorded on a AV 250 spectrometers or a Bruker Avance NEO 400MHz NanoBay spectrometer at the Faculté de Pharmacie of Marseille or on a Bruker Avance III nanobay 400 MHz spectrometer at the Spectropole, Faculté des Sciences de Saint-Jêrome (Marseille). ($^1$H NMR: reference $CHCl_3$ δ = 7.26 ppm, reference DMSO-$d_6$ δ = 2.50 ppm and $^{13}$C NMR: reference $CHCl_3$ δ = 76.9 ppm, reference DMSO-$d_6$ δ = 39.52 ppm). The following adsorbent was used for column chromatography: silica gel 60 (Merck KGaA, Darmstadt, Germany, particle size 0.063–0.200 mm, 70–230 mesh ASTM). TLC was performed on 5 cm × 10 cm aluminum plates coated with silica gel 60F-254 (Merck) in an appropriate eluent. Visualization was performed with ultraviolet light (234 nm). Purity of synthesized compounds was checked by LC/MS analyses, which were realized at the Faculté de Pharmacie of Marseille with a Thermo Scientific Accela High Speed LC System®® (Waltham, MA, USA) coupled using a single quadrupole mass spectrometer Thermo MSQ Plus®®. The RP-HPLC column is a Thermo Hypersil Gold®® 50 × 2.1 mm (C18 bounded), with particles of a diameter of 1.9 mm. The volume of sample injected on the column was 1 μL. Chromatographic analysis, total duration of 8 min, was on the gradient of the following solvents: t = 0 min, methanol/water 50:50; 0 < t < 4 min, linear increase in the proportion of methanol to a methanol/water ratio of 95:5; 4 < t < 6 min, methanol/water 95:5; 6 < t < 7 min, linear decrease in the proportion of methanol to return to a methanol/water ratio of 50:50; 6 < t < 7 min, methanol/water 50:50. The water used was buffered with ammonium acetate 5 mM. The flow rate of the mobile phase was 0.3 mL/min. The retention times (tR) of the molecules analyzed were indicated in min. High-resolution MS experiments were performed at the Spectropole (Campus Etoile, Aix-Marseille Université, France) with a SYNAPT G2 HDMS quadrupole/time-of-flight (Q/ToF) (Waters, Manchester, UK) using the following parameters: ESI capillary voltage: +2.8 kV; extraction cone voltage: 20 V; and desolvation gas flow rate (nitrogen): 100 L·h$^{-1}$. The accurate mass measurements were carried out in triplicate with an external calibration. Reagents were purchased and used without further purifications from Sigma-Aldrich or Fluorochem. The starting product 2-nitropyrrole (**1**) was prepared using previous reports.[16]

**3.1.2 Synthesis of Ethyl 2-(2-nitro-1*H*-pyrrol-1-yl)acetate (2)**

To a solution of 2-nitropyrrole (0.5 g, 4.76 mmol) in EtOH (20 mL) was added $K_2CO_3$ (0.679 g, 4.91 mmol, 1.1 eq) and ethyl bromoacetate (0.544 ml, 4.91 mmol, 1.1 eq). The reaction mixture was heated at 70 °C for 12 h. After EtOH evaporation, 20 ml of water was poured on the crude product and the aqueous layer was extracted with dichloromethane (3 × 50 mL). The organic layer was washed with brine (3 × 100 mL), dried over $Na_2SO_4$ and evaporated. The crude product was purified by column chromatography (silica gel, petroleum ether /ethyl acetate 9:1).

Yield 71% (629 mg). White solid. Mp 51-52 °C.

$^1$H NMR (400 MHz, $CDCl_3$) δ 7.26-7.24 (m, 1H, CH), 6.81 (dd, $^3J_{H-H}$ = 2.8 Hz, $^4J_{H-H}$ = 2.1 Hz, 1H, CH), 6.25 (dd, $^3J_{H-H}$ = 4.4 Hz, $^4J_{H-H}$ = 2.8 Hz, 1H, CH), 5.02 (s, 2H, $CH_2$), 4.24 (q, $^3J_{H-H}$ = 7.1 Hz, 2H, $CH_2$), 1.28 (t, $^3J_{H-H}$ = 7.1 Hz, 3H, $CH_3$).

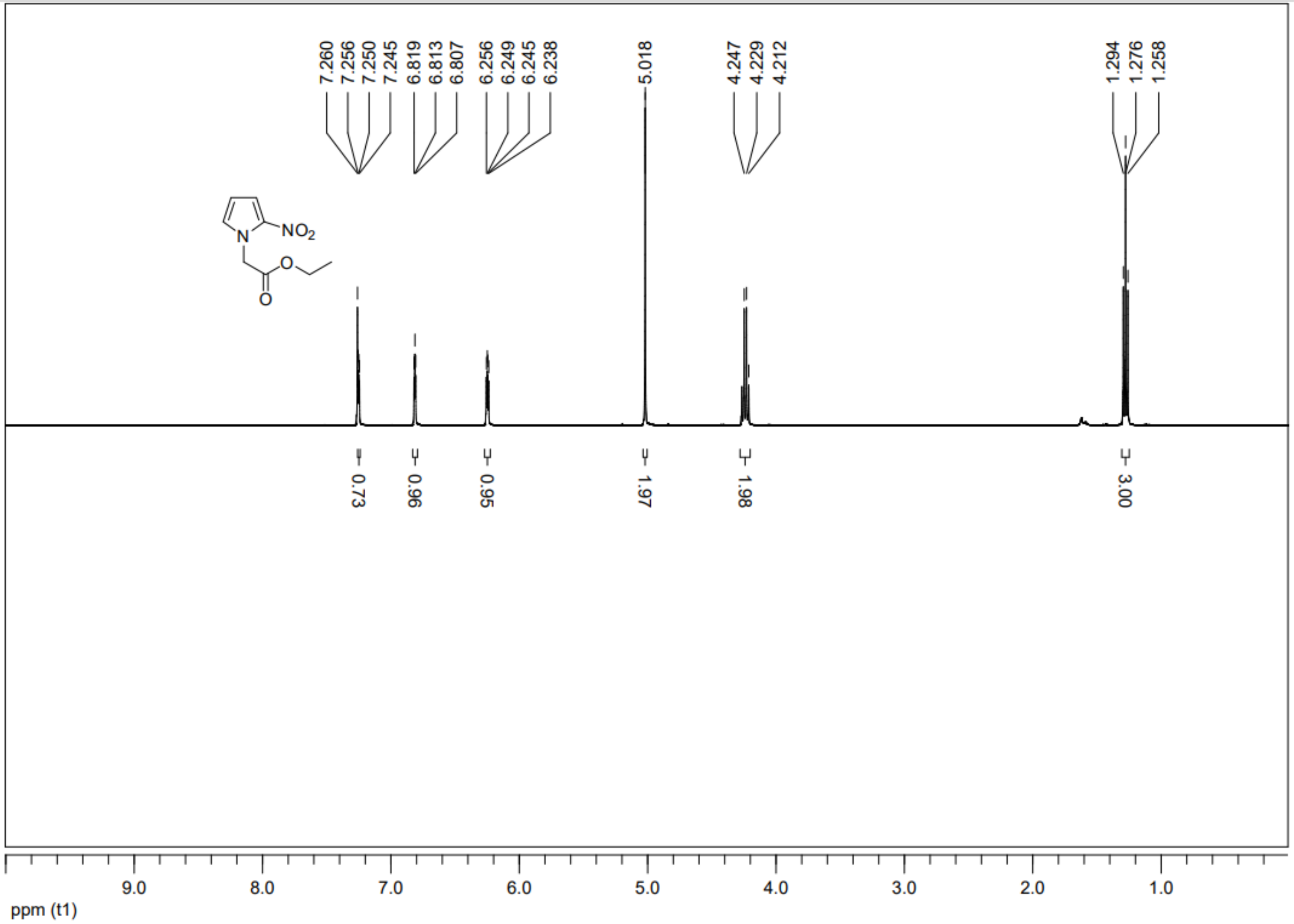

$^{13}C$ NMR (100 MHz, $CDCl_3$) δ 167.5 (C), 137.7 (C), 130.0 (CH), 114.9 (CH), 109.2 (CH), 62.2 ($CH_2$), 51.7 ($CH_2$), 14.2 ($CH_3$).

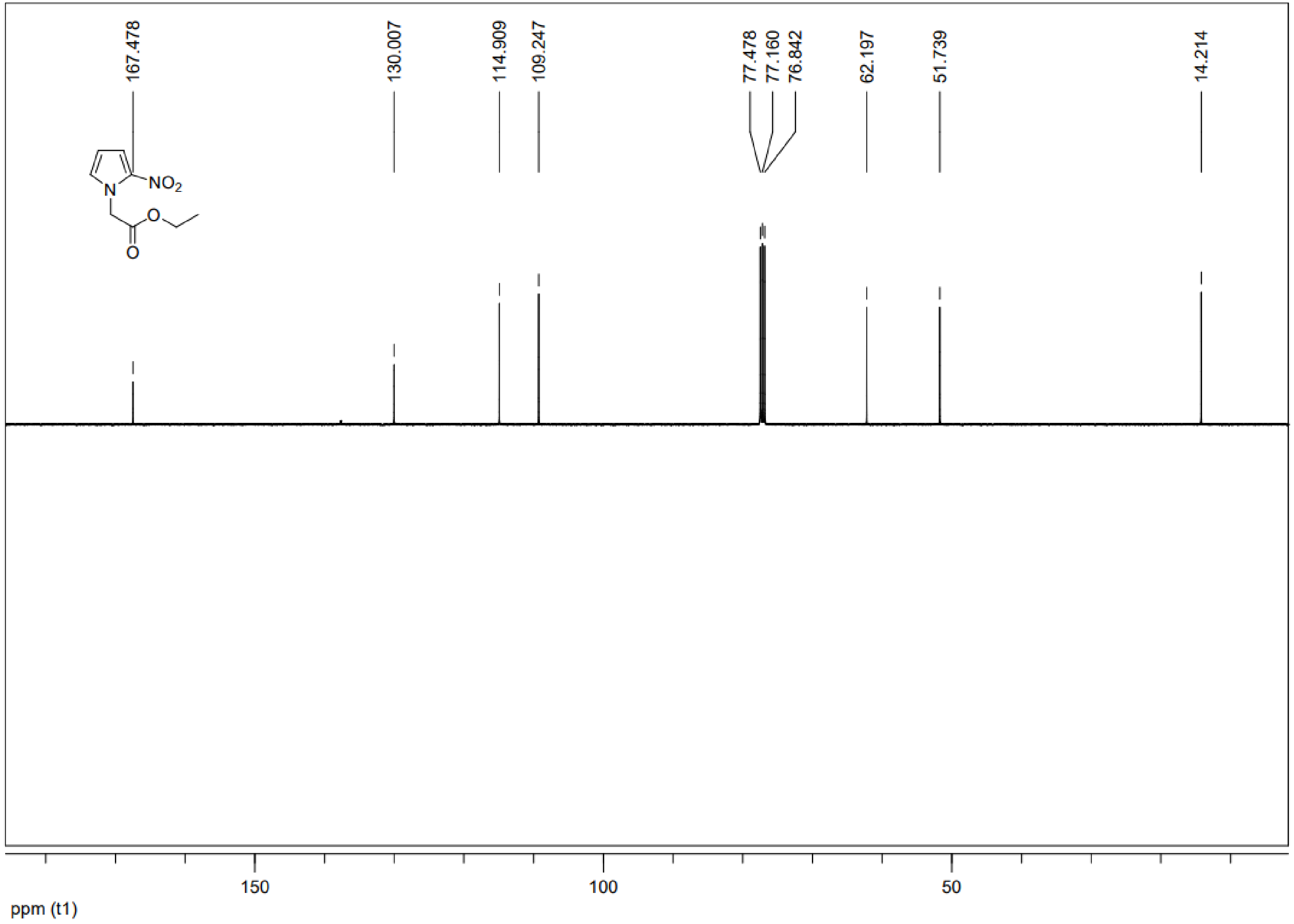

### 3.1.3 General procedure for the synthesis of bromoacetamide intermediate derivatives

In a first vial, dichloromethane (10 mL) was added to bromoacetyl bromide (216 µL, 2.48 mmol, 1 eq). In a second vial, dichloromethane (10 mL) was added to the corresponding amine (2.48 mmol, 1 eq) and $Et_3N$ (345 µL, 2.48 mmol, 1 eq). The two reactions mixtures were respectively stirred for 5 min at 0 °C in a bath of ice. The second solution mixture was added dropwise at 0 °C to the first bromoacetyl bromide solution and the reaction mixture was stirred from 0 °C to room temperature for 12 h. The solution was poured into a bath of ice. The aqueous layer was extracted with dichloromethane (3 × 100 mL) and the organic layer was washed with brine (3 × 100 mL), dried over $Na_2SO_4$ and evaporated. Intermediate compounds were obtained without purification excepted for compounds **7, 10, 11, 14.**

**$N$-Benzyl-2-bromoacetamide (5)**[24]

Yield 82% (460 mg). White solid. Lit. Mp 106-107.5 °C.[25]

$^1$H NMR (400 MHz, DMSO-d6) δ 8.78 (s, 1H, NH), 7.35–7.31 (m, 2H, 2CH), 7.27-7.25 (m, 3H, 3CH), 4.30 (d, $^3J_{H-H}$ = 6.0 Hz, 2H, $CH_2$), 3.91 (s, 2H, $CH_2$). The $^1$H NMR data were in agreement with the literature values. Lit. $^{13}$C NMR (125 MHz, $CDCl_3$) δ 165.3 (C),137.2 (C), 128.8 (2CH), 127.8 (CH), 127.7 (2CH), 44.2 ($CH_2$), 29.1 ($CH_2$).

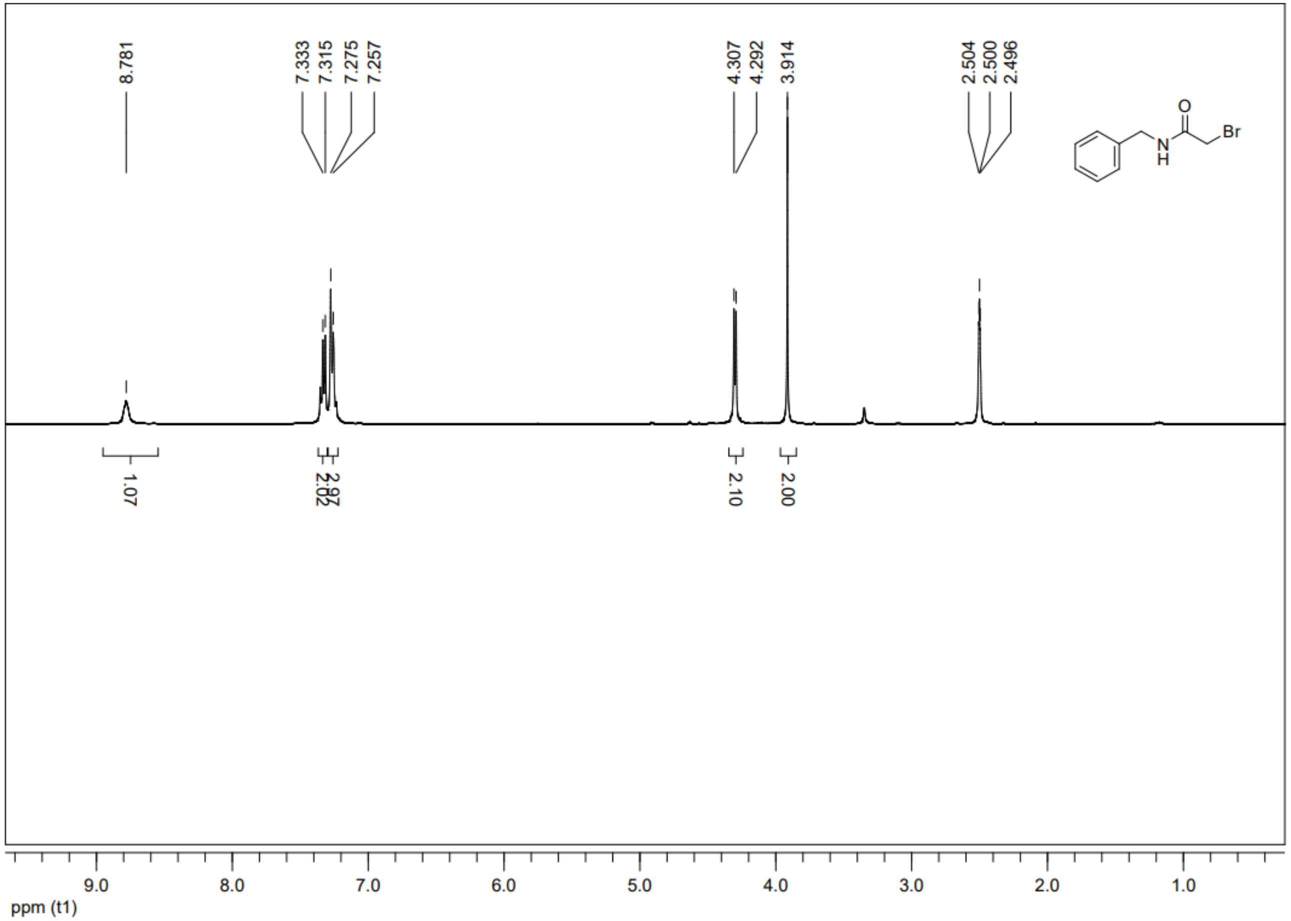


24. Xie, H.; Ng, D.; Savinov, S.N.; Dey, B.; Kwong, P.D.; Wyatt, R.; Smith, A.B.; Hendrickson, W.A. Structure−Activity Relationships in the Binding of Chemically Derivatized CD4 to Gp120 from Human Immunodeficiency Virus. *J. Med. Chem.* **2007**, *50*, 4898–4908.

25. Kushner, S.; Cassell, R.I.; Morton, J.; Williams, J.H. Anticonvulsants. N-Benzylamides. *J. Org. Chem.* **1951**, *16*, 1283–1288.

**2-Bromo-*N*-phenylacetamide (6)** [24]-[26]

Yield 99% (525 mg). Brown solid. Lit. Mp 129-131 °C (isopropyl ether).[26] $^{1}$H NMR (400 MHz, DMSO-d6) δ 10.37 (s, 1H, NH), 7.60–7.57 (m, 2H, 2CH), 7.35-7.30 (m, 2H, 2CH), 7.10-7.07 (m, 1H, CH), 4.04 (s, 2H, $CH_2$). The $^{1}$H NMR data were in agreement with the literature values. Lit. $^{13}$C NMR (125 MHz, $CDCl_3$) δ 163.3 (C), 136.9 (C), 129.1 (2CH), 125.2 (2CH), 120.0 (CH), 29.5 ($CH_2$).

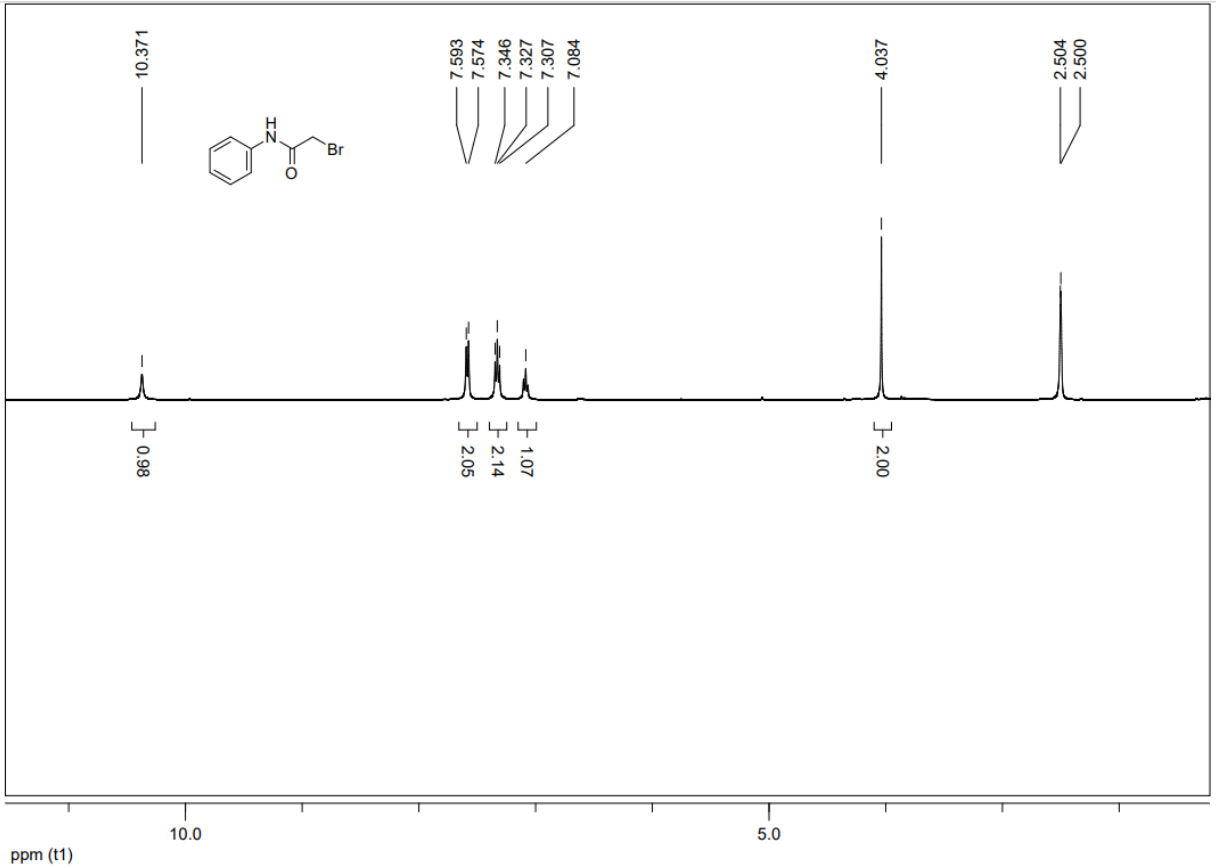



24. Xie, H.; Ng, D.; Savinov, S.N.; Dey, B.; Kwong, P.D.; Wyatt, R.; Smith, A.B.; Hendrickson, W.A. Structure−Activity Relationships in the Binding of Chemically Derivatized CD4 to Gp120 from Human Immunodeficiency Virus. *J. Med. Chem.* **2007**, *50*, 4898–4908.
26. Ferraccioli, R.; Forni, A. Selective Synthesis of Isoquinolin-3-One Derivatives Combining Pd-Catalysed Aromatic Alkylation/Vinylation with Addition Reactions: The Beneficial Effect of Water. *Eur. J. Org. Chem.* **2009**, *2009*, 3161–3166.

**2-Bromo-*N*-[3-(trifluoromethyl)benzyl]acetamide (7)[27]**

The crude product was purified by column chromatography [silica gel, dichloromethane/petroleum ether from 2/8 to 8/2). Yield 18% (130 mg). Brown solid. Lit. Mp 251-253 °C.[28] $^{1}$H NMR (400 MHz, $CDCl_3$) δ 7.57-7.53 (m, 2H, 2CH), 7.49-7.47 (m, 2H, 2CH), 6.86 (s, 1H, NH), 4.54 (d, $^{3}J_{H\text{-}H}$ = 6.1 Hz, 2H, $CH_2$), 3.95 (s, 2H, $CH_2$). The $^{1}$H NMR data were in agreement with the literature values. Lit. $^{13}$C NMR ($CDCl_3$, 75 MHz) δ 165.6 (C), 141.4 (C), 130.0 (q, $^{3}J_{C\text{-}F}$ = 32.3 Hz, C), 127.8 (2CH), 125.7 (q, $^{4}J_{C\text{-}F}$ = 3.8 Hz, CH), 122.1 (CH), 118.6 (C), 43.6 ($CH_2$), 28.9 ($CH_2$).

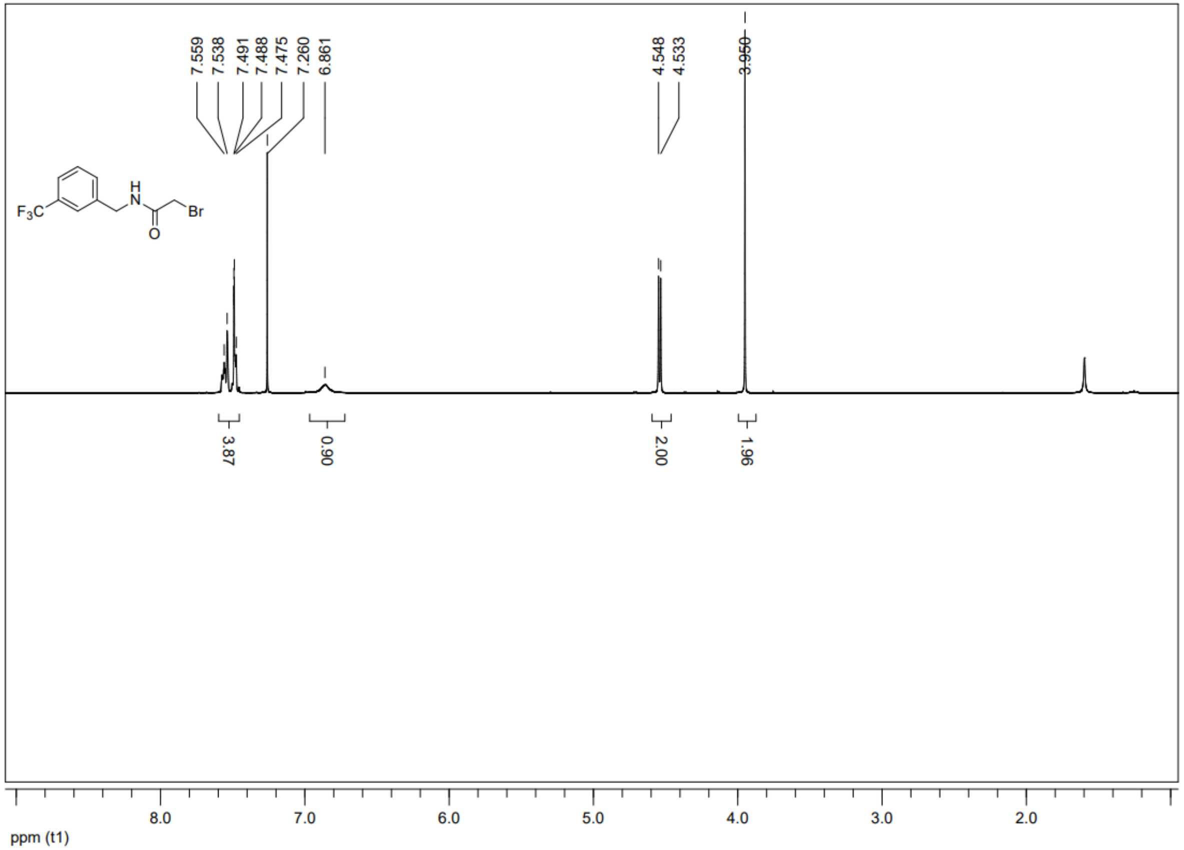



27. Kadjane, P.; Platas-Iglesias, C.; Boehm-Sturm, P.; Truffault, V.; Hagberg, G.E.; Hoehn, M.; Logothetis, N.K.; Angelovski, G. Dual-Frequency Calcium-Responsive MRI Agents. *Chem. Eur. J.* **2014**, *20*, 7351–7362.
28. Jogula, S.; Krishna, V.S.; Meda, N.; Balraju, V.; Sriram, D. Design, Synthesis and Biological Evaluation of Novel Pseudomonas Aeruginosa DNA Gyrase B Inhibitors. *Bioorg. chem.* **2020**, *100*, 103905.

**2-Bromo-*N*-(4-bromophenyl)acetamide (8)** [29]

Yield 91% (657 mg). Brown solid. Lit. Mp 148-150 °C. $^{1}$H NMR (400 MHz, DMSO-d6) δ 10.51 (s, 1H, NH), 7.57-7.50 (m, 4H, 4CH), 4.03 (s, 2H, $CH_2$). The $^{1}$H NMR data were in agreement with the literature values. Lit. $^{13}$C NMR ($CDCl_3$, 100 MHz,) δ 163.3 (C), 136.0 (C), 132.1 (2CH), 121.5 (2CH), 117.9 (C), 29.3 ($CH_2$).

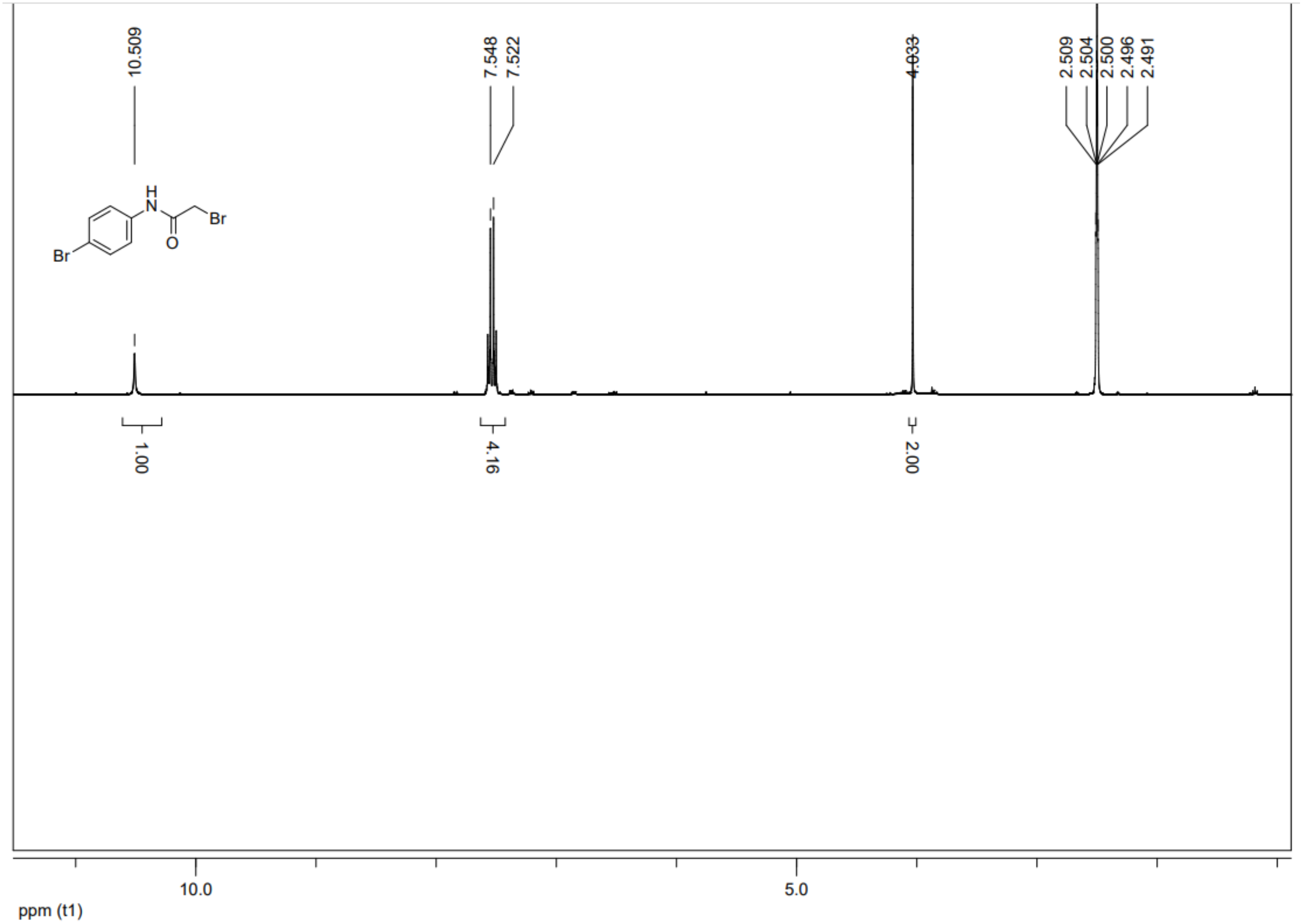



29. Jiang, C.; Shi, J.; Liao, L.; Zhang, L.; Liu, J.; Wang, Y.; Lao, Y.; Zhang, J. 5-[2-(N-(Substituted Phenyl)Acetamide)]Amino-1,3,4-thiadiazole-2-sulfonamides as Selective Carbonic Anhydrase II Inhibitors with Neuroprotective Effects. *ChemMedChem* **2020**, *15*, 705–715.

**2-Bromo-*N*-(2-chlorophenyl)acetamide (9)**

Yield 44% (657 mg). Brown solid. Mp 92-93 °C. $^{1}H$ NMR (400 MHz, DMSO-d6) δ 9.94 (s, 1H, NH), 7.71 (dd, $^{3}J_{H-H}$ = 6.1 Hz, $^{4}J_{H-H}$ = 1.7 Hz, 1H, CH), 7.52 (dd, $^{3}J_{H-H}$ = 8.0 Hz, $^{4}J_{H-H}$ = 1.5 Hz, 1H, CH), 7.35 (td, $^{3}J_{H-H}$ = 7.8 Hz, $^{4}J_{H-H}$ = 1.5 Hz, 1H, CH), 7.23 (td, $^{3}J_{H-H}$ = 7.7 Hz, $^{4}J_{H-H}$ = 1.7 Hz, 1H, CH), 4.17 (s, 2H, $CH_2$).

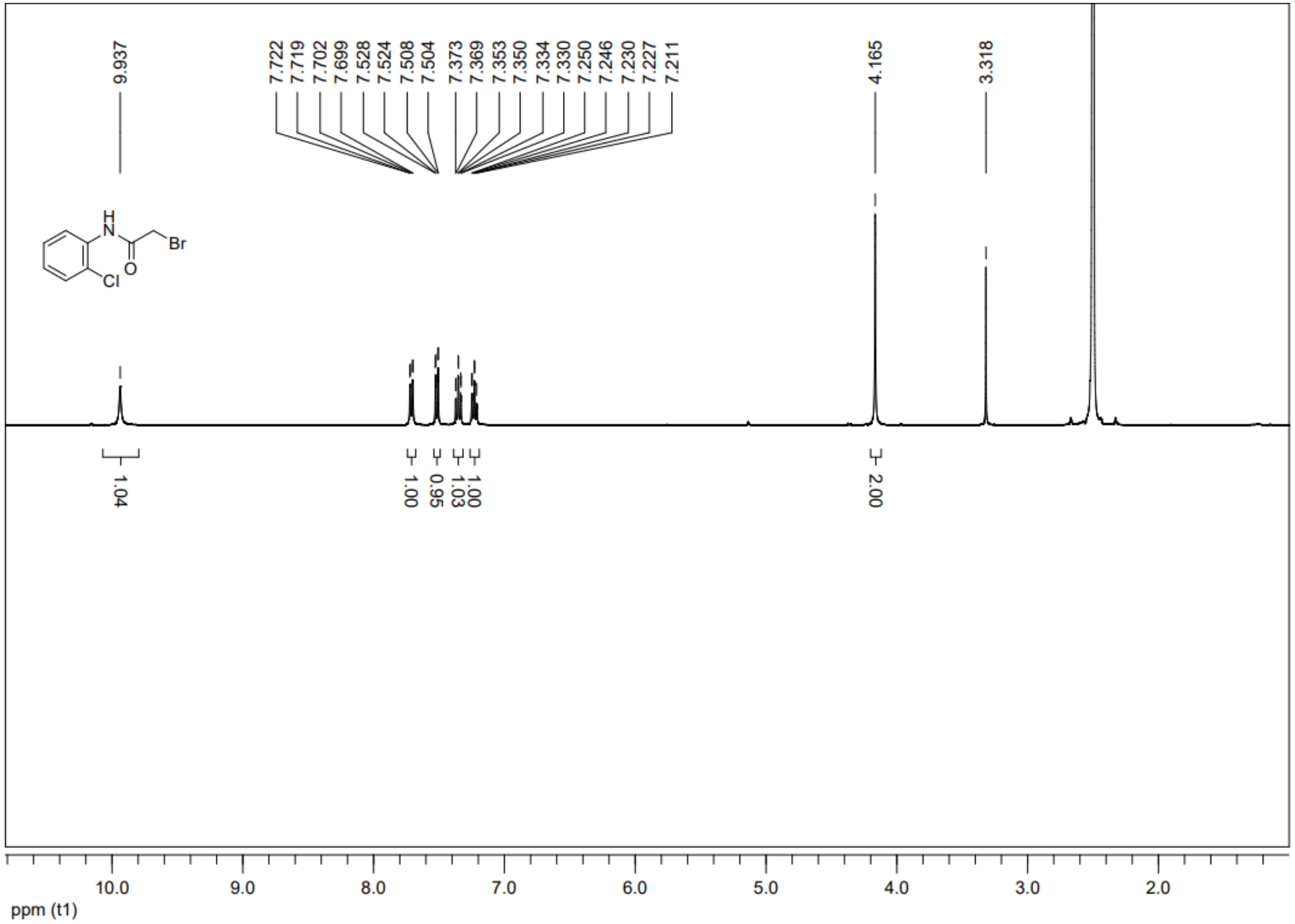

$^{13}C$ NMR (100 MHz, DMSO-d6) δ 170.7 (C), 134.2 (C), 129.3 (CH), 127.9 (2CH), 125.3 (CH), 122.0 (C), 61.6 ($CH_2$).

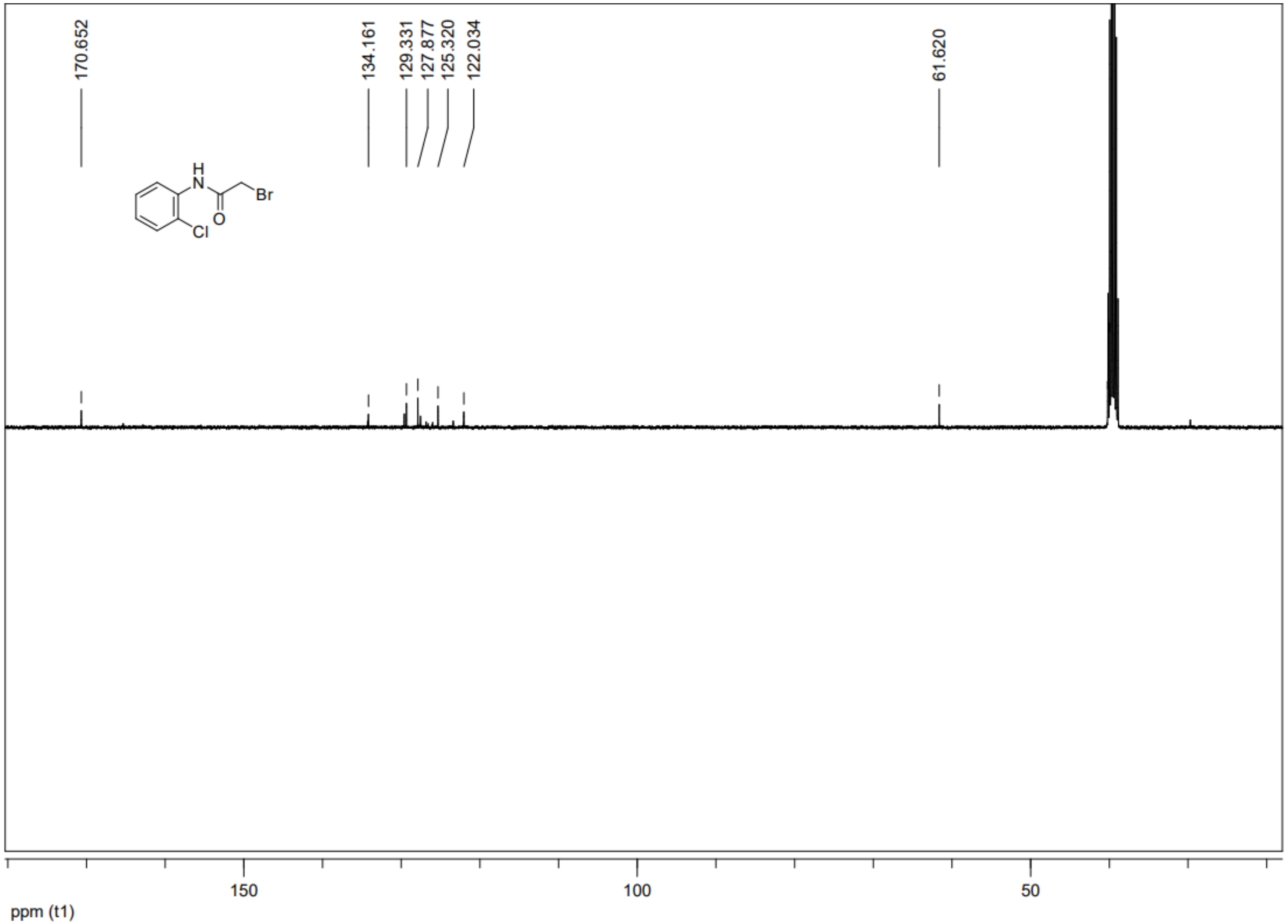

**2-Bromo-*N*-[3-(trifluoromethyl)phenyl]acetamide (10)[28]**

The crude product was purified by column chromatography [silica gel, dichloromethane/petroleum ether 6/4). Yield 48% (331 mg). White solid. Lit. Mp 78–81 °C. $^{1}$H NMR (400 MHz, DMSO-d6) δ 10.72 (s, 1H, NH), 8.07 (s, 1H, CH), 7.76 (d, $^{3}J_{H\text{-}H}$ = 8.4 Hz, 1H, CH), 7.60-7.56 (m, 1H, CH), 7.44 (d, $^{3}J_{H\text{-}H}$ = 7.6 Hz, 1H, CH), 4.07 (s, 2H, $CH_2$). The $^{1}$H NMR data were in agreement with the literature values. Lit. $^{13}$C NMR (100 MHz, $CDCl_3$): δ 173.7 (C), 138.4 (C), 130.9 (C), 130.6 (CH), 127.8 (CH), 124.6 (C), 123.5 (CH), 123.1 (CH), 42.7 ($CH_2$).

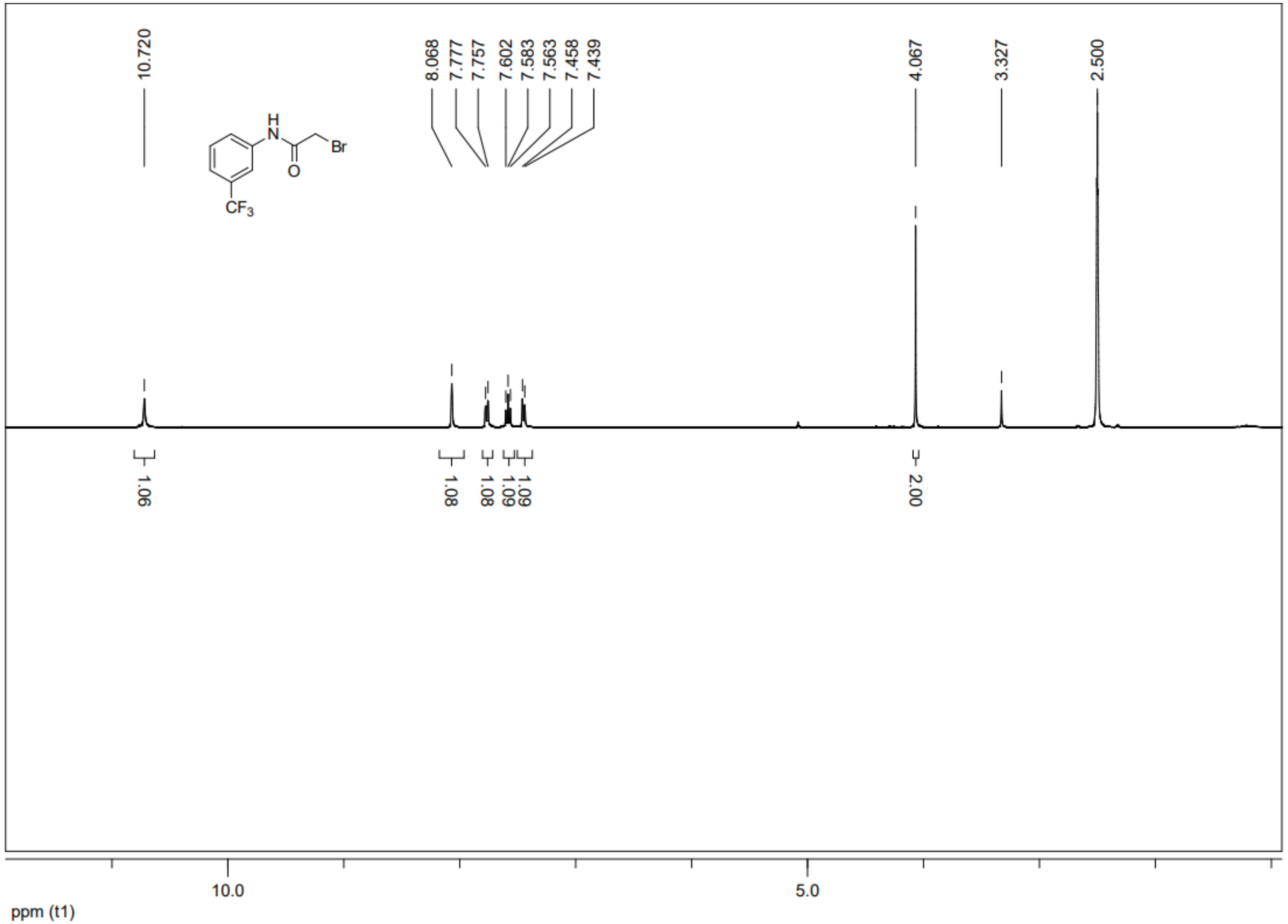



28. Jogula, S.; Krishna, V.S.; Meda, N.; Balraju, V.; Sriram, D. Design, Synthesis and Biological Evaluation of Novel Pseudomonas Aeruginosa DNA Gyrase B Inhibitors. *Bioorg. chem.* **2020**, *100*, 103905.

**2-Bromo-*N*-(3-methoxyphenyl)acetamide (11)[30]**

The crude product was purified by column chromatography [silica gel, dichloromethane/petroleum ether 6/4). Yield 46% (280 mg). White solid. Mp 94-95 °C. $^1$H NMR (400 MHz, $CDCl_3$) δ 8.10 (s, 1H, NH), 7.27-7.26 (m, 1H, CH), 7.25-7.23 (m, 1H, CH), 7.02-6.99 (m, 1H, CH), 6.73-6.71 (m, 1H, CH), 4.02 (s, 2H, $CH_2$), 3.81 (s, 3H, $CH_3$). The $^1$H NMR data were in agreement with the literature values. Lit. $^{13}$C NMR (63 MHz, DMSO-d6) δ 164.7 (C), 159.5 (C), 139.7 (C), 129.6 (CH), 111.5 (CH), 109.2 (CH), 105.0 (CH), 55.0 ($CH_3$), 30.4 ($CH_2$).

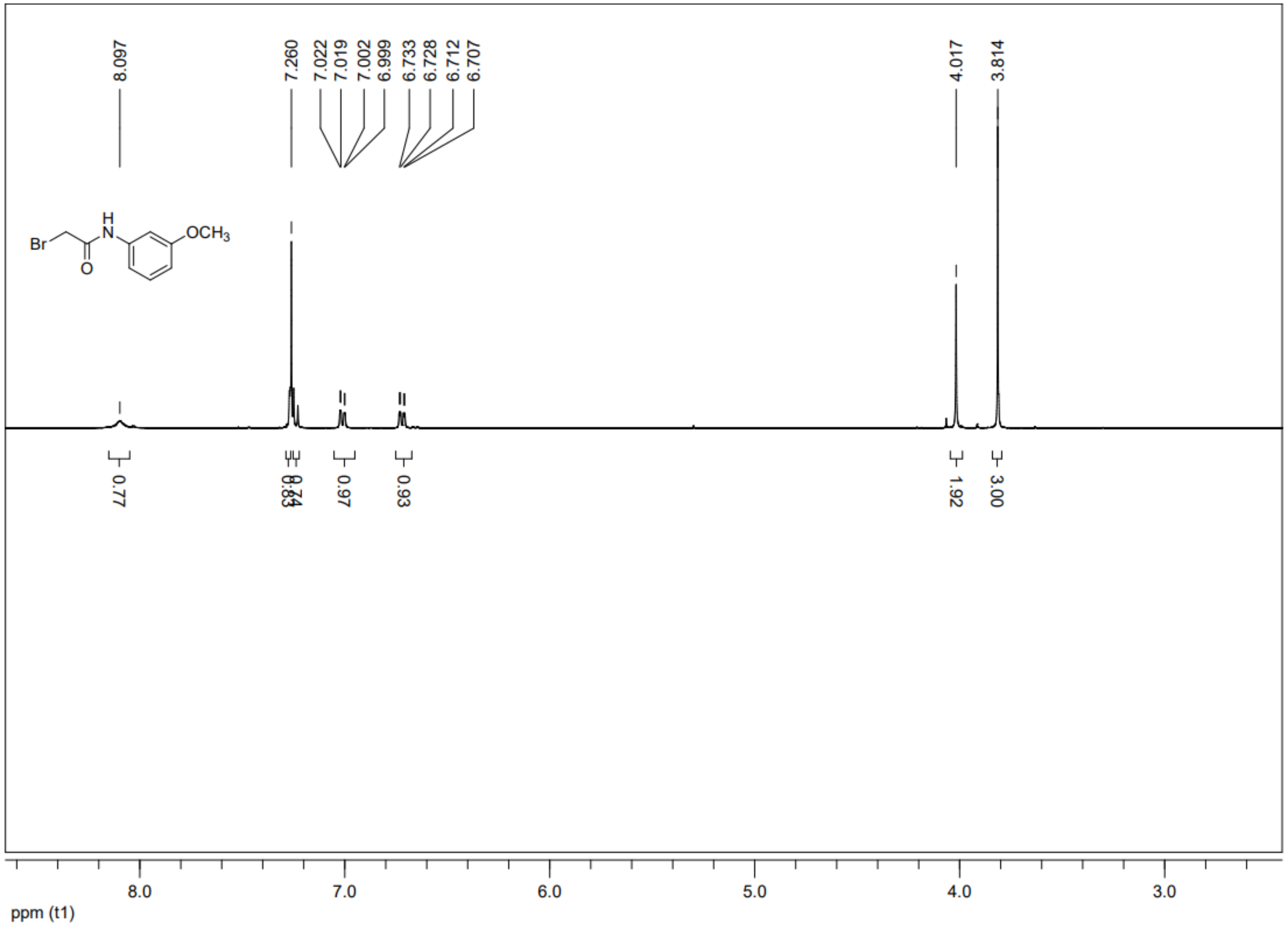


30. Samanta, S.; Lim, T.L.; Lam, Y. Synthesis and in Vitro Evaluation of West Nile Virus Protease Inhibitors Based on the 2-{6-[2-(5-Phenyl-4 *H* -{1,2,4]Triazol-3-Ylsulfanyl)Acetylamino]Benzothiazol-2-Ylsulfanyl}acetamide Scaffold. *ChemMedChem* **2013**, *8*, 994–1001.

**2-Bromo-*N*-isopropylacetamide (12)**[31]

Yield 25% (100 mg). White solid. Mp Lit. 63-64 °C. $^1$H NMR (400 MHz, $CDCl_3$) δ 6.29 (s, 1H, NH), 4.10-4.02 (m, 1H, CH), 3.85 (s, 2H, $CH_2$), 1.19 (d, $^3J_{H\text{-}H}$ = 6.5 Hz, 6H, $2CH_3$). The $^1$H NMR data were in agreement with the literature values. Lit. $^{13}$C NMR (400 MHz, $CDCl_3$) δ 164.4 (C), 42.3 (CH), 29.4 ($CH_2$), 22.4 ($2CH_3$).

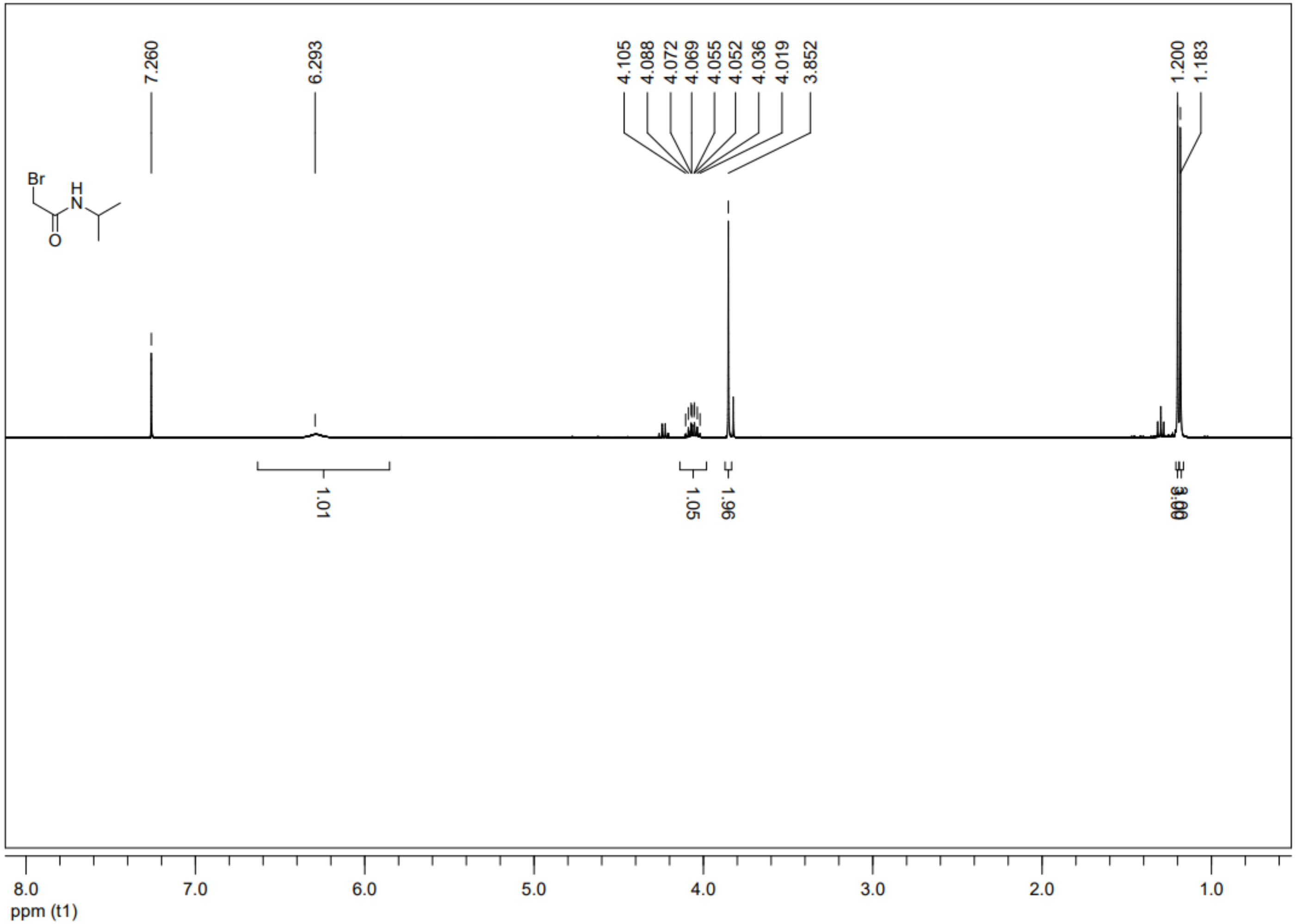


31. Lucas, R.L.; Zart, M.K.; Murkerjee, J.; Sorrell, T.N.; Powell, D.R.; Borovik, A.S. A Modular Approach toward Regulating the Secondary Coordination Sphere of Metal Ions: Differential Dioxygen Activation Assisted by Intramolecular Hydrogen Bonds. *J. Am. Chem. Soc.* **2006**, *128*, 15476–15489.

**2-Bromo-*N*-cyclopentylacetamide (13)[31]**

Yield 87% (443 mg). White solid. Mp Lit. 84-85 °C. $^{1}H$ NMR (400 MHz, $CDCl_3$) δ = 6.74 (s, 1H, NH), 4.13-4.08 (m, 1H, CH), 3.78 (s, 2H, $CH_2$), 1.94-1.87 (m, 2H, $CH_2$), 1.65-1.63 (m, 2H, $CH_2$), 1.56-1.53 (m, 2H, $CH_2$), 1.40-1.36 (m, 2H, $CH_2$). The $^{1}H$ NMR data were in agreement with the literature values. Lit. $^{13}C$ NMR (400 MHz, $CDCl_3$) δ = 164.8 (C), 51.8 (CH), 32.8 ($2CH_2$), 29.4 ($CH_2$), 23.6 ($2CH_2$).

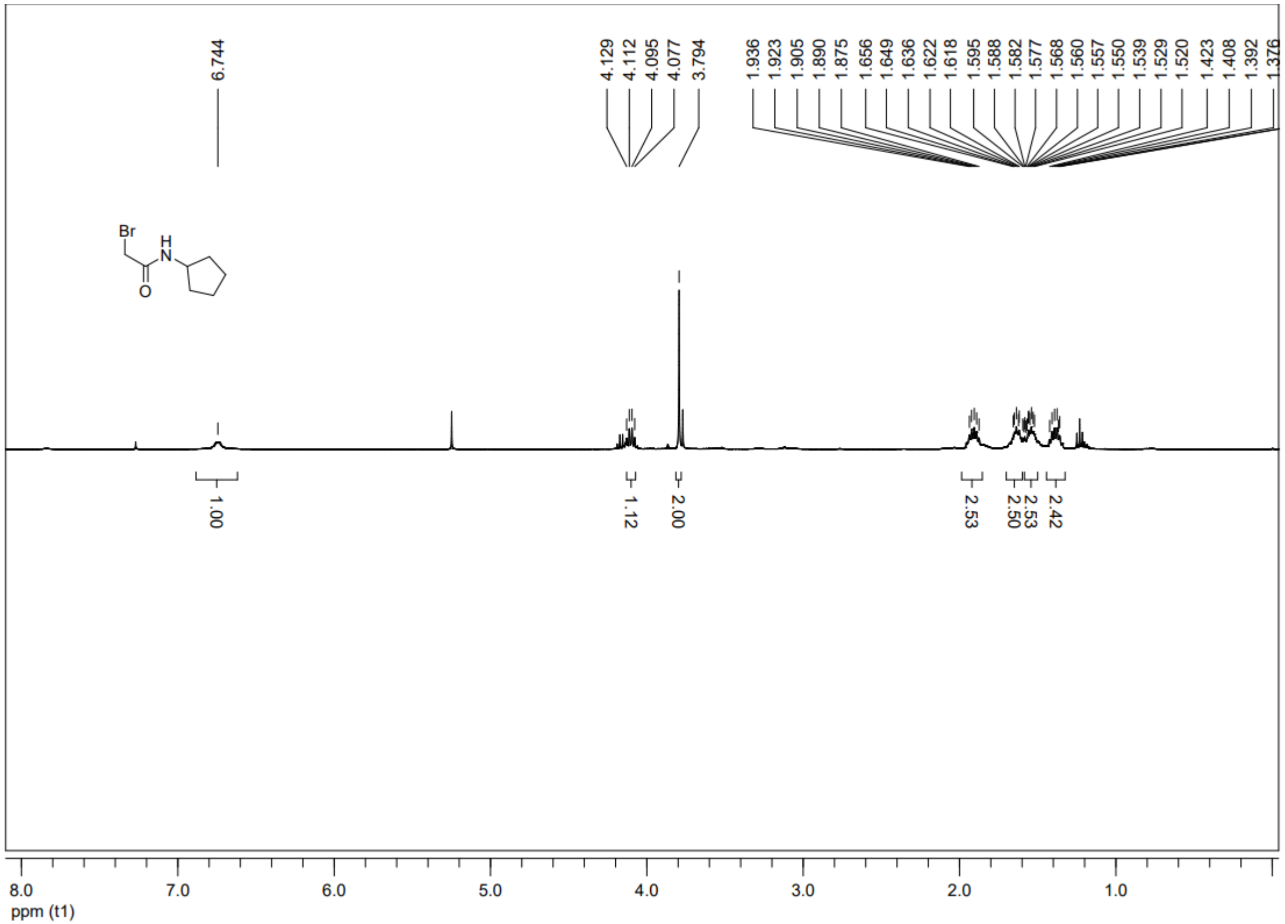



31. Lucas, R.L.; Zart, M.K.; Murkerjee, J.; Sorrell, T.N.; Powell, D.R.; Borovik, A.S. A Modular Approach toward Regulating the Secondary Coordination Sphere of Metal Ions: Differential Dioxygen Activation Assisted by Intramolecular Hydrogen Bonds. *J. Am. Chem. Soc.* **2006**, *128*, 15476–15489.

**2-Bromo-*N*-(2-bromo-5-fluorophenyl)acetamide (14)**

The crude product was purified by column chromatography [silica gel, dichloromethane/petroleum ether 5/5). Yield 25% (280 mg). White solid. Mp. 99-100 °C. $^1$H NMR (400 MHz, $CDCl_3$) δ 8.86 (s, 1H, NH), 8.24-8.21 (m, 1H, CH), 7.53-7.50 (m, 1H, CH), 6.81-6.76 (m, 1H, CH), 4.08 (s, 2H, $CH_2$).

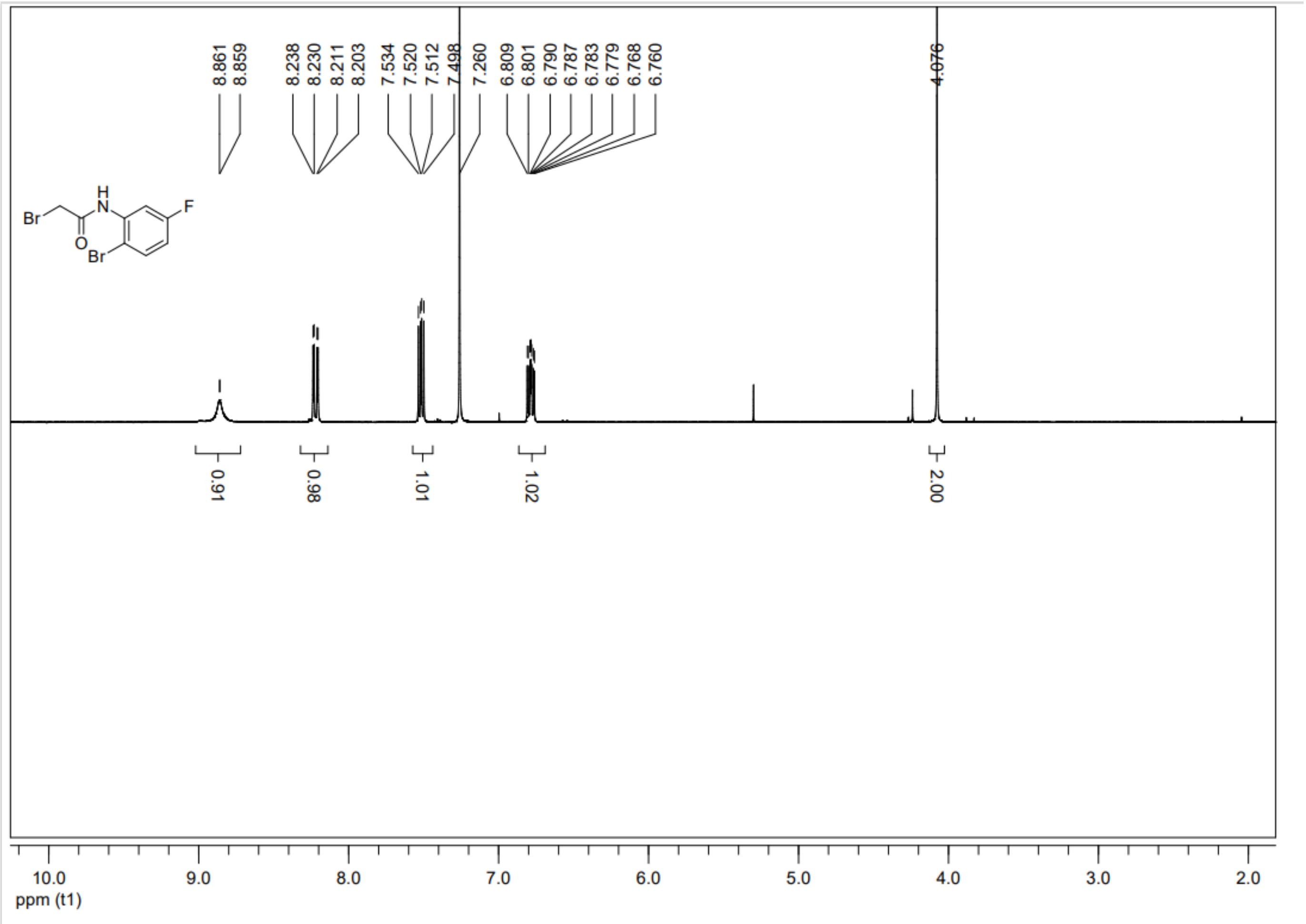

$^{13}C$ NMR (100 MHz, $CDCl_3$) δ 163.7 (C), 162.1 (d, $^{1}J_{C-F}$ = 246.3 Hz, C), 136.3 (d, $^{3}J_{C-F}$ = 11.6 Hz, C), 133.1 (d, $^{3}J_{C-F}$ = 9.5 Hz, CH), 113.0 (d, $^{2}J_{C-F}$ = 23.2 Hz, CH), 108.9 (d, $^{2}J_{C-F}$ = 29.1 Hz, CH), 107.7 (d, $^{4}J_{C-F}$ = 3.6 Hz, C), 29.7 ($CH_2$).

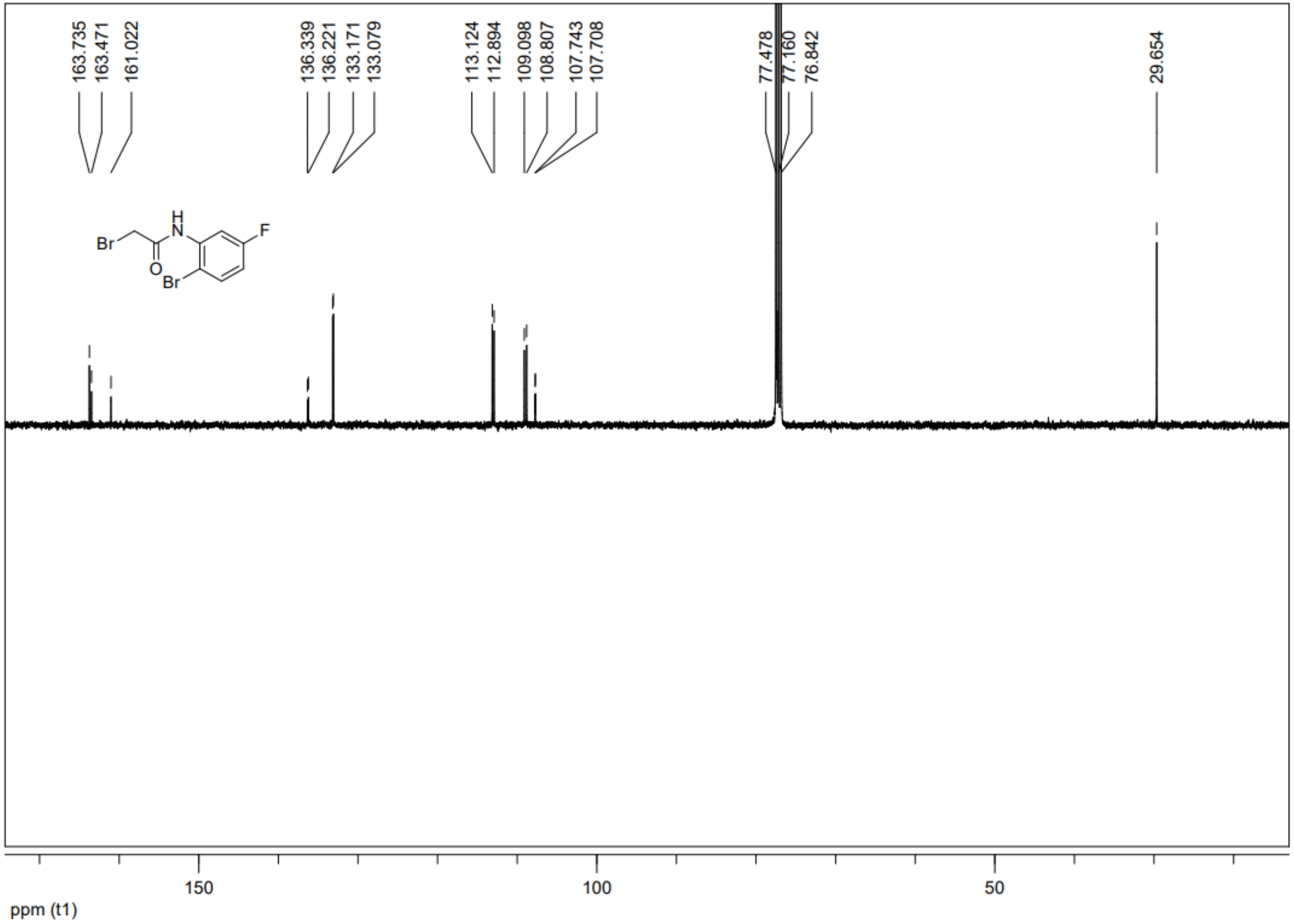

### 3.1.4 General procedure for the synthesis of 2-nitropyrrole derivatives

To a solution of 2-nitropyrrole (0.1 g, 0.9 mmol, 1 eq) in ethanol (5 mL), was added $K_2CO_3$ (0.138 g, 0.99 mmol, 1.1 eq) and the corresponding bromoacetamide compound (1.1 eq). The reaction mixture was stirred and heated at 70 °C for 3 h. After cooling, the solution was poured into a bath of ice. A precipitate appeared and was filtered, washed with water (3 × 100 mL), petroleum ether (3 × 100 mL) and dried in vacuum drying oven (dessicator cabinet).

***N*-Benzyl-2-(2-nitro-1*H*-pyrrol-1-yl)acetamide (3)**

Yield 73% (113 mg). White solid. Mp 174-175 °C.

LC-MS (ESI+) Tr 4.12 min, m/z $[M + H]^+$ 260.18. MW: 259.10 g.mol$^{-1}$. HRMS: m/z $[M + H]^+$ calcd for $[C_{13}H_{13}N_3O_3]^+$ : 260.1030 ; found: 260.1029.

$^{1}H$ NMR (400 MHz, DMSO-d6) δ 8.70 (s, 1H, NH), 7.33-7.25 (m, 7H, 7CH), 6.28 (dd, $^{3}J_{H\text{-}H}$ = 4.4 Hz, $^{4}J_{H\text{-}H}$ =2.7 Hz, 1H, CH), 5.09 (s, 2H, $CH_2$), 4.32 (d, $^{3}J_{H\text{-}H}$ = 5.9 Hz, 2H, $CH_2$).

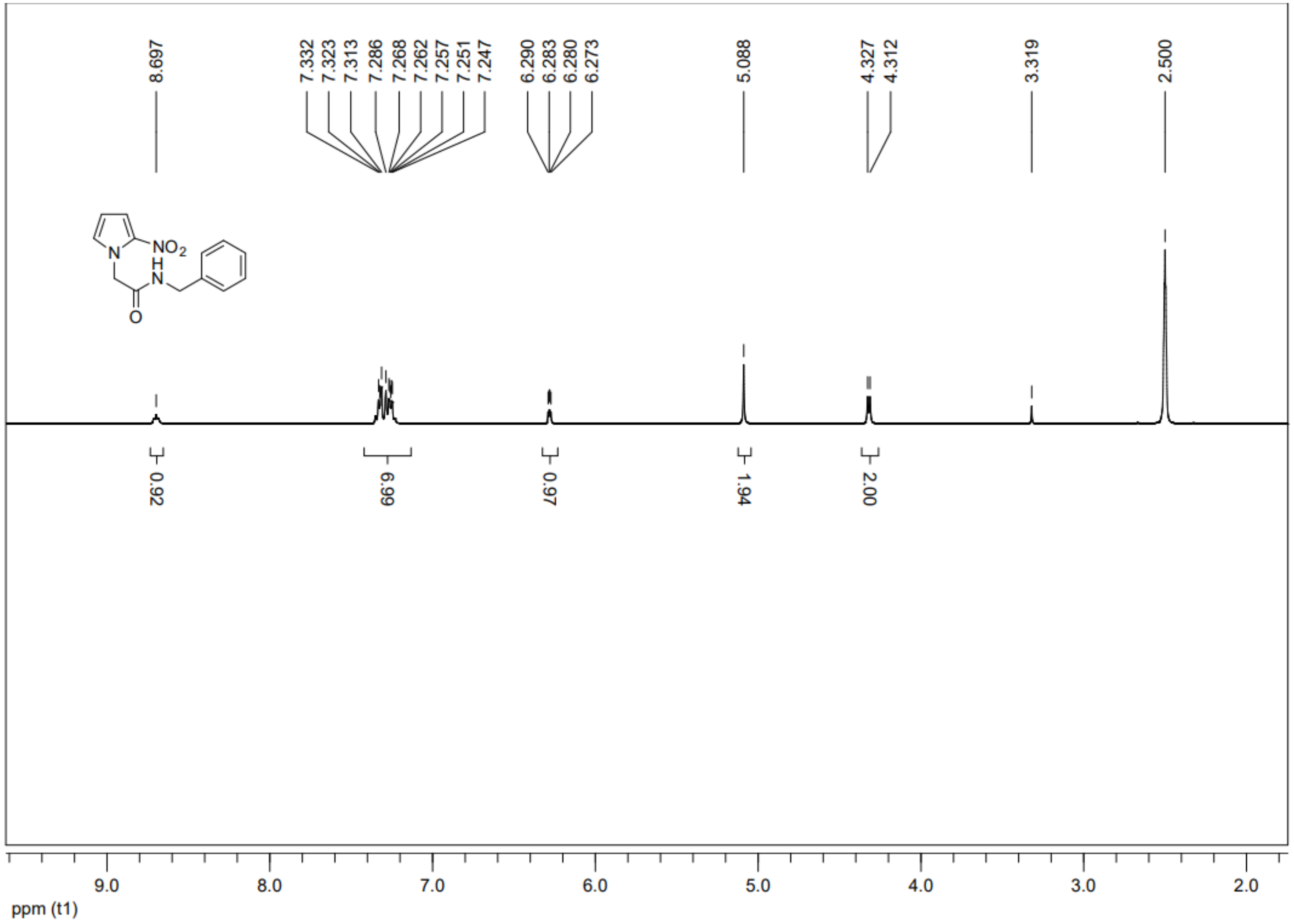

$^{13}C$ NMR (100 MHz, DMSO-d6) δ 167.1 (C), 139.5 (C), 137.6 (C), 132.9 (CH), 128.7 (2CH), 127.7 (2CH), 127.3 (CH), 114.8 (CH), 109 (CH), 52.6 ($CH_2$), 42.7 ($CH_2$).

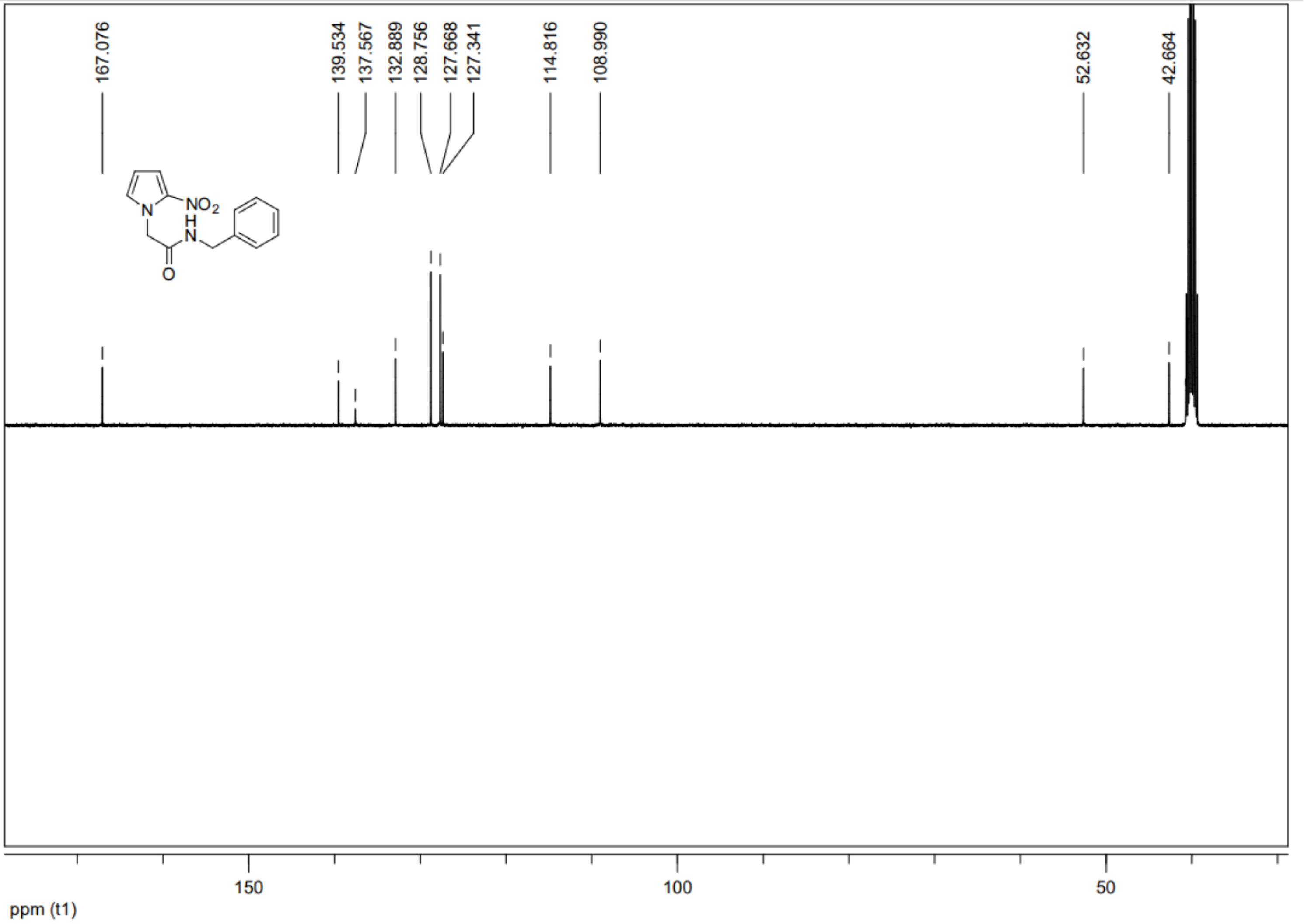

**2-(2-Nitro-1*H*-pyrrol-1-yl)-*N*-phenylacetamide (4)**

Yield 78% (170 mg). White solid. Mp 201-202 °C.

LC-MS (ESI+) Tr 4.08 min, m/z $[M + H]^+$ 246.21. MW: 245.08 g.mol$^{-1}$. HRMS: m/z $[M + H]^+$ calcd for $[C_{12}H_{11}N_3O_3]^+$ : 246.0873 ; found: 246.0873.

$^{1}$H NMR (400 MHz, DMSO-d6) δ 10.4 (s, 1H, NH), 7.57-7.55 (m, 2H, 2CH), 7.36-7.28 (m, 4H, 4CH), 7.06 (t, $^3J_{H\text{-}H}$ = 7.4 Hz, 1H, CH), 6.32 (dd, $^3J_{H\text{-}H}$ = 4.4 Hz, $^4J_{H\text{-}H}$ = 2.7 Hz, 1H, CH), 5.24 (s, 2H, $CH_2$).

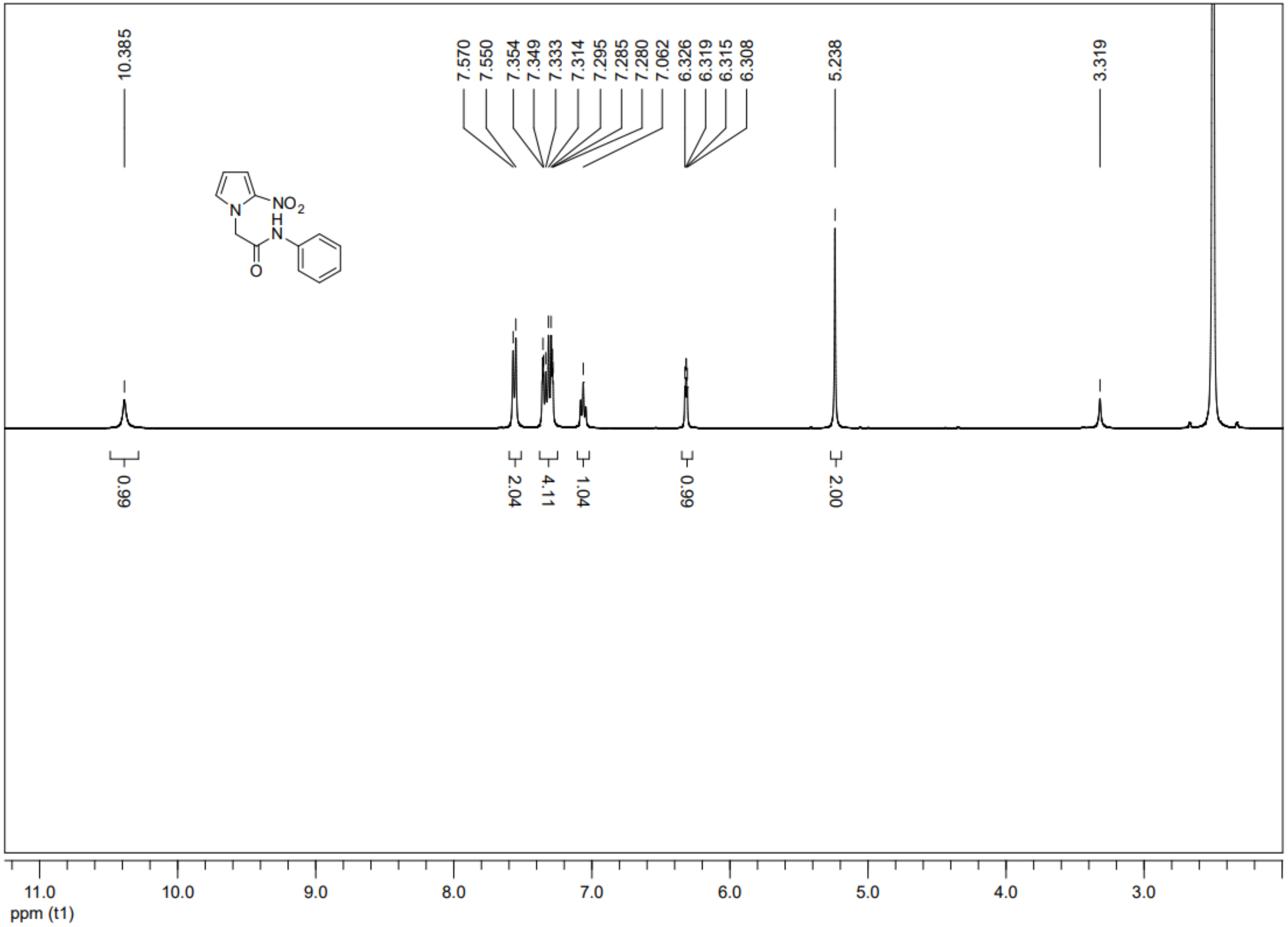

$^{13}C$ NMR (100 MHz, DMSO-d6) δ 165.3 (C), 138.7 (C), 137.0 (C), 132.5 (CH), 128.9 (2CH), 123.5 (CH), 119.0 (2CH), 114.4 (CH), 108.6 (CH), 52.8 ($CH_2$).

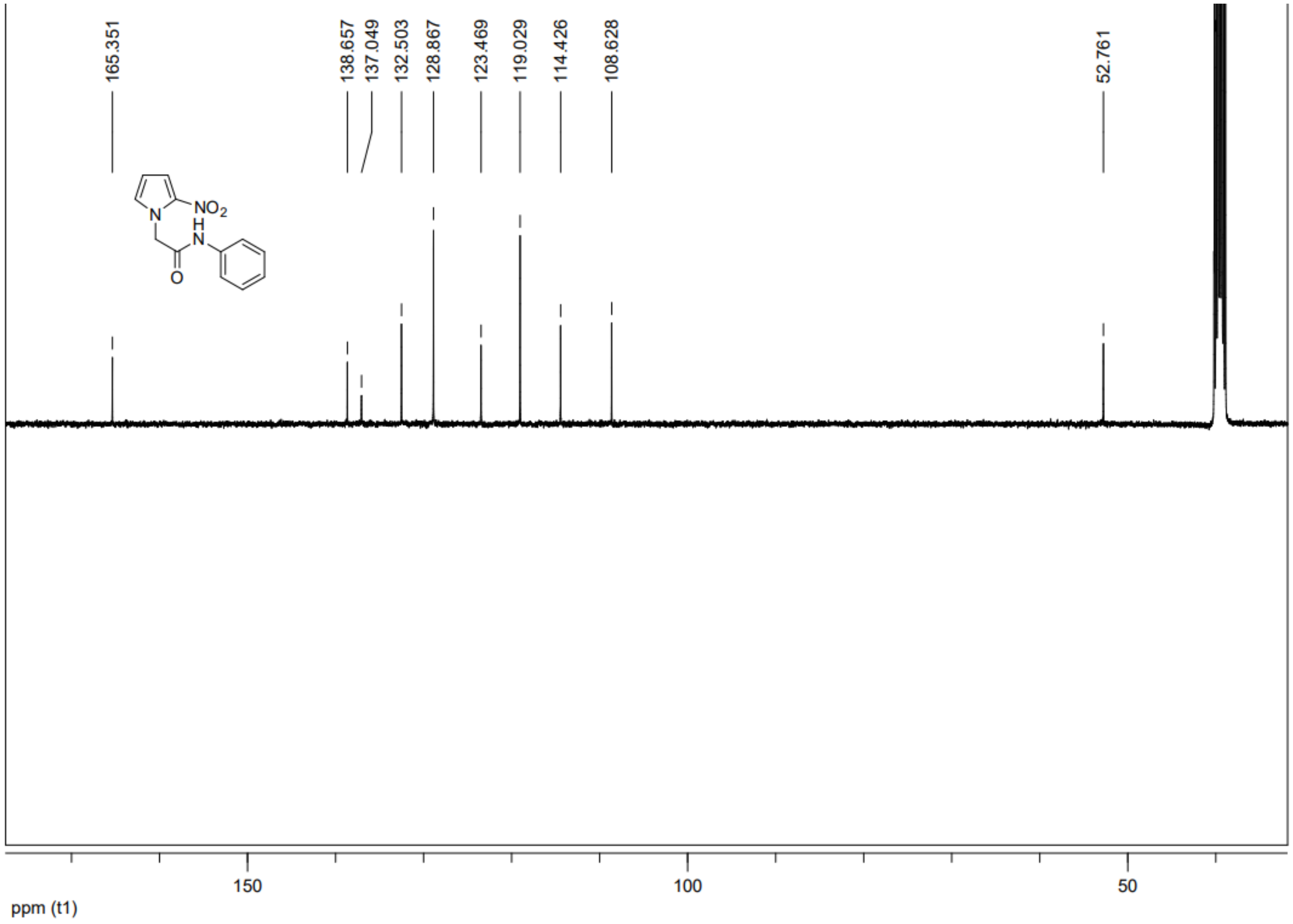

**2-(2-Nitro-1*H*-pyrrol-1-yl)-*N*-[3-(trifluoromethyl)benzyl]acetamide (15)**

Yield 60% (172 mg). White solid. Mp 161-162 °C. LC-MS (ESI+) Tr 5.07 min, m/z $[M + H]^+$ 328.09. MW: 327.08g.mol$^{-1}$. HRMS: m/z $[M + H]^+$ calcd for $[C_{14}H_{12}F_3N_3O_3]^+$ : 328.0904 ; found: 328.0901.

$^{1}$H NMR (400 MHz, DMSO-d6) δ 8.81 (s, 1H, NH), 7.64-7.57 (m, 4H, 4CH), 7.33-7.30 (m, 1H, CH), 7.25 (dd, $^{3}J_{H\text{-}H}$ = 4.1 Hz, $^{4}J_{H\text{-}H}$ = 1.9 Hz 1H, CH), 6.28 (dd, $^{3}J_{H\text{-}H}$ = 3.8 Hz, $^{4}J_{H\text{-}H}$ = 2.7 Hz, 1H, CH), 5.11 (s, 2H, $CH_2$), 4.42 (d, $^{3}J_{H\text{-}H}$ = 5.8 Hz, 2H, $CH_2$).

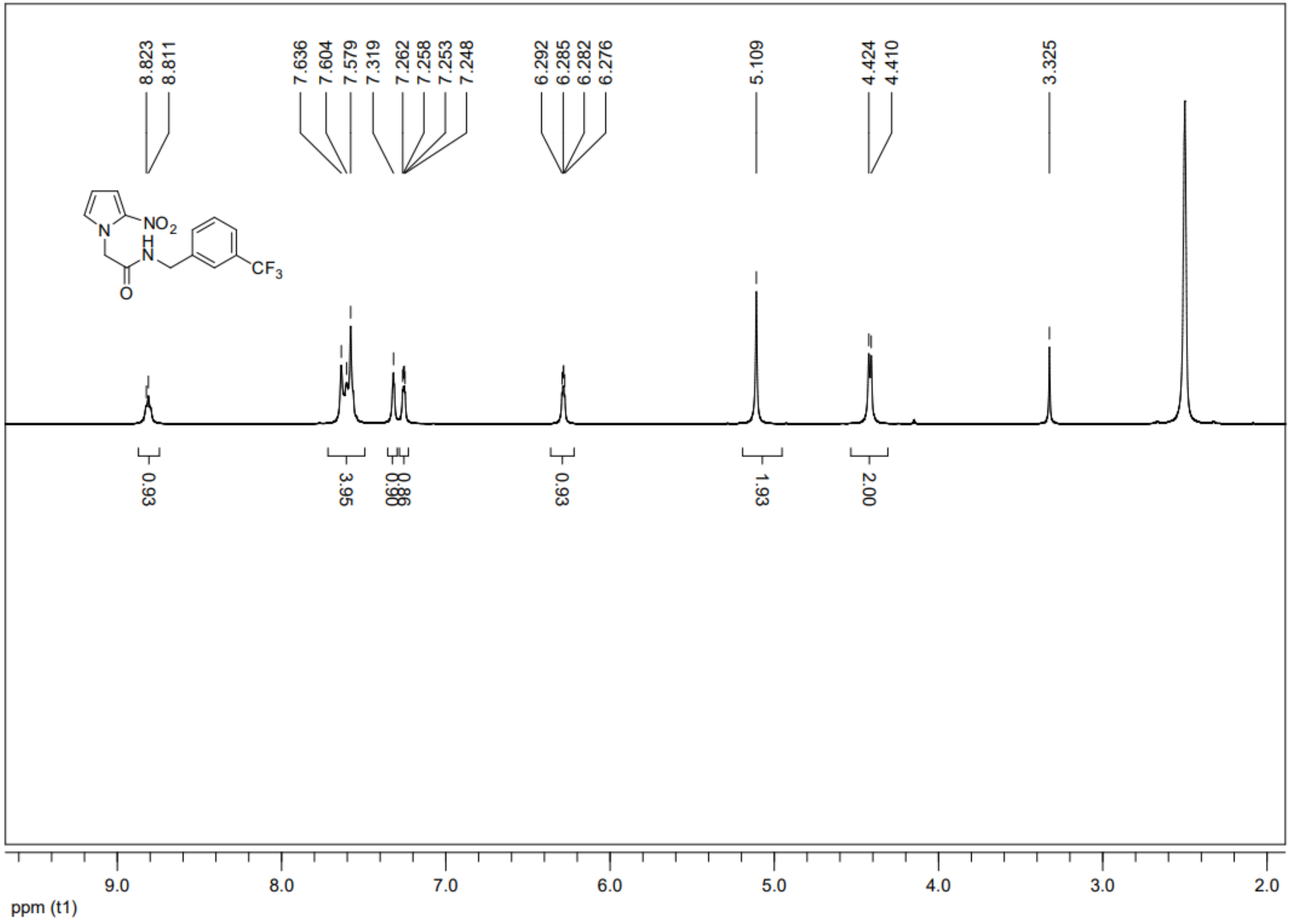

$^{13}C$ NMR (100 MHz, DMSO-d6) δ 167.0 (C), 140.7 (C), 137.1 (C), 132.4 (CH), 131.2 (CH), 129.3 (CH), 129.1 (q, $^{2}J_{C-F}$ = 31.8 Hz, C), 124.2 (q, $^{1}J_{C-F}$ = 272.4 Hz, C), 123.6 (q, $^{3}J_{C-F}$ = 3,8 Hz, 2CH), 114.3 (CH), 108.5 (CH), 52.2 ($CH_2$), 41.7 ($CH_2$).

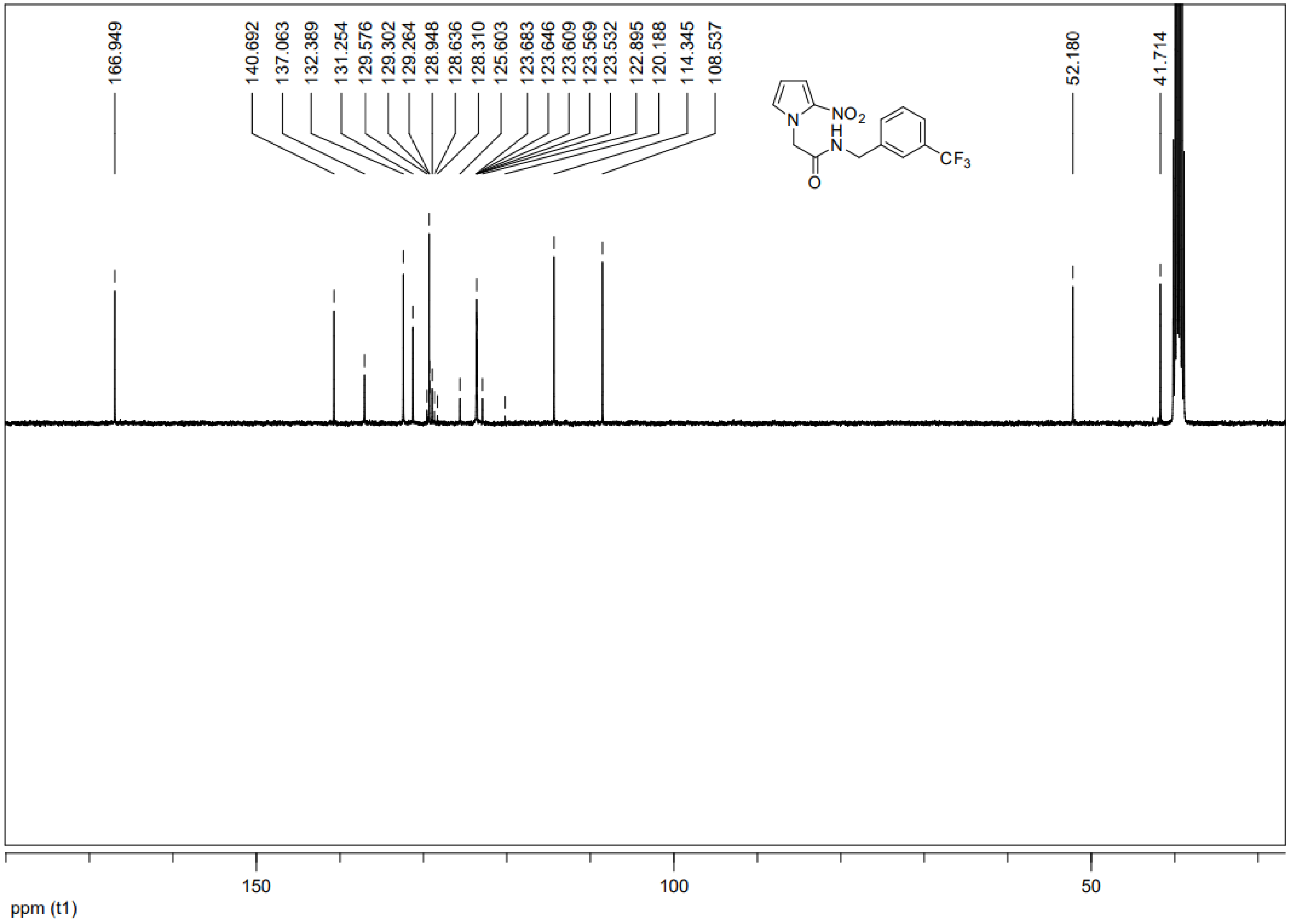

**_N_-(4-Bromophenyl)-2-(2-nitro-1_H_-pyrrol-1-yl)acetamide (16)**

Yield 86% (291 mg). White solid. Mp 229-230 °C. LC-MS (ESI+) Tr 5.05 min, m/z $[M + H]^+$ 323.98. MW: 322.99 $g.mol^{-1}$. HRMS: m/z [M + H]+ calcd for $[C_{12}H_{10}BrN_3O_3]^+$ : 323.9978 ; found: 323.9977.

$^{1}H$ NMR (400 MHz, DMSO-d6) δ 10.52 (s, 1H, NH), 7.55-7.52 (m, 4H, 4CH), 7.35-7.34 (m, 1H, CH), 7.34-7.30 (m, 1H, CH), 6.32 (dd, $^{3}J_{H\text{-}H}$ = 3.8 Hz, $^{4}J_{H\text{-}H}$ = 2.8 Hz, 1H, CH), 5.24 (s, 2H, $CH_2$).

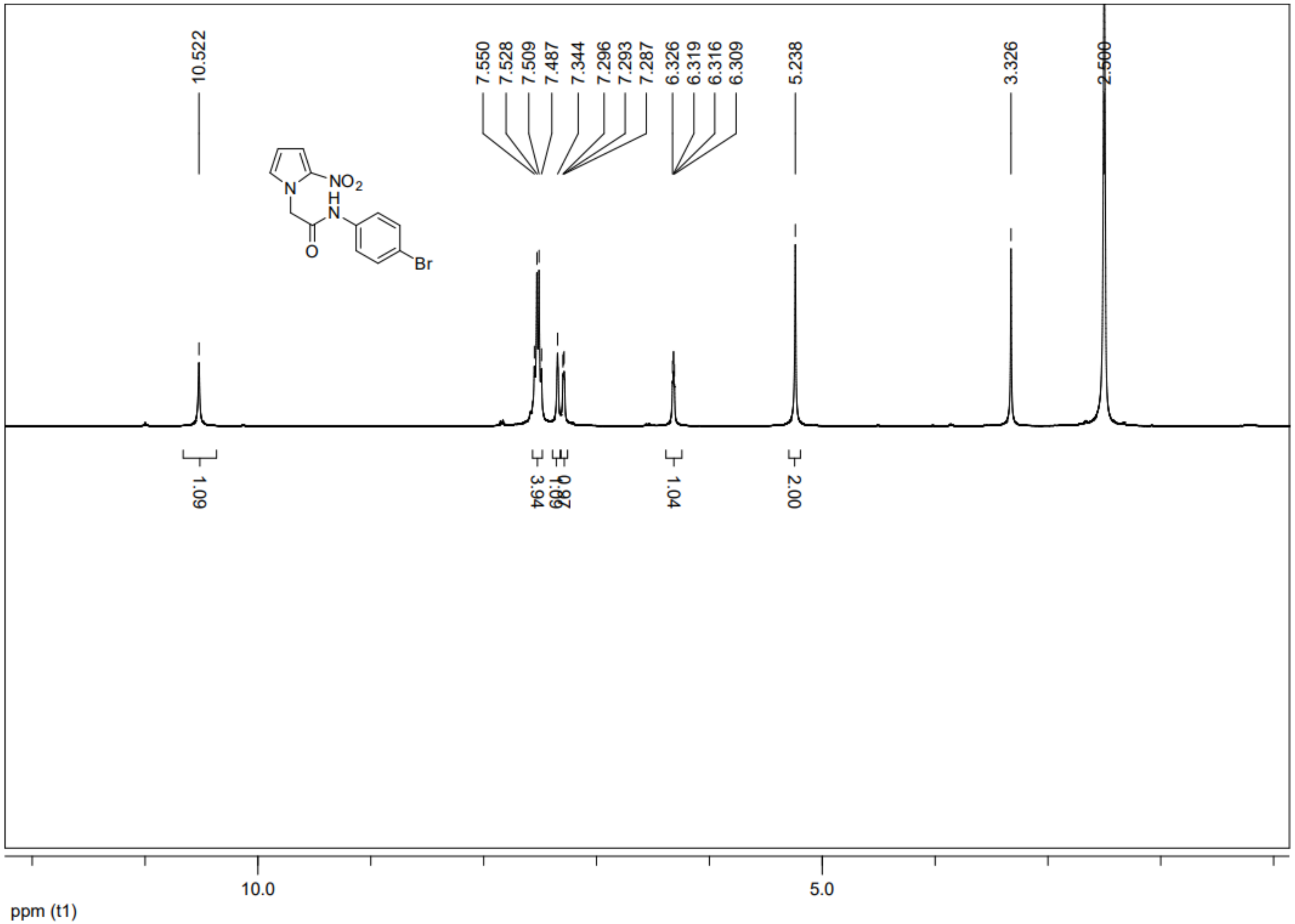

$^{13}C$ NMR (100 MHz, DMSO-d6) δ 165.6 (C), 138.0 (C), 137.0 (C), 132.5 (CH), 131.7 (2CH), 121.0 (2CH), 115.1 (C), 114.1 (CH), 108.7 (CH), 52.8 ($CH_2$).

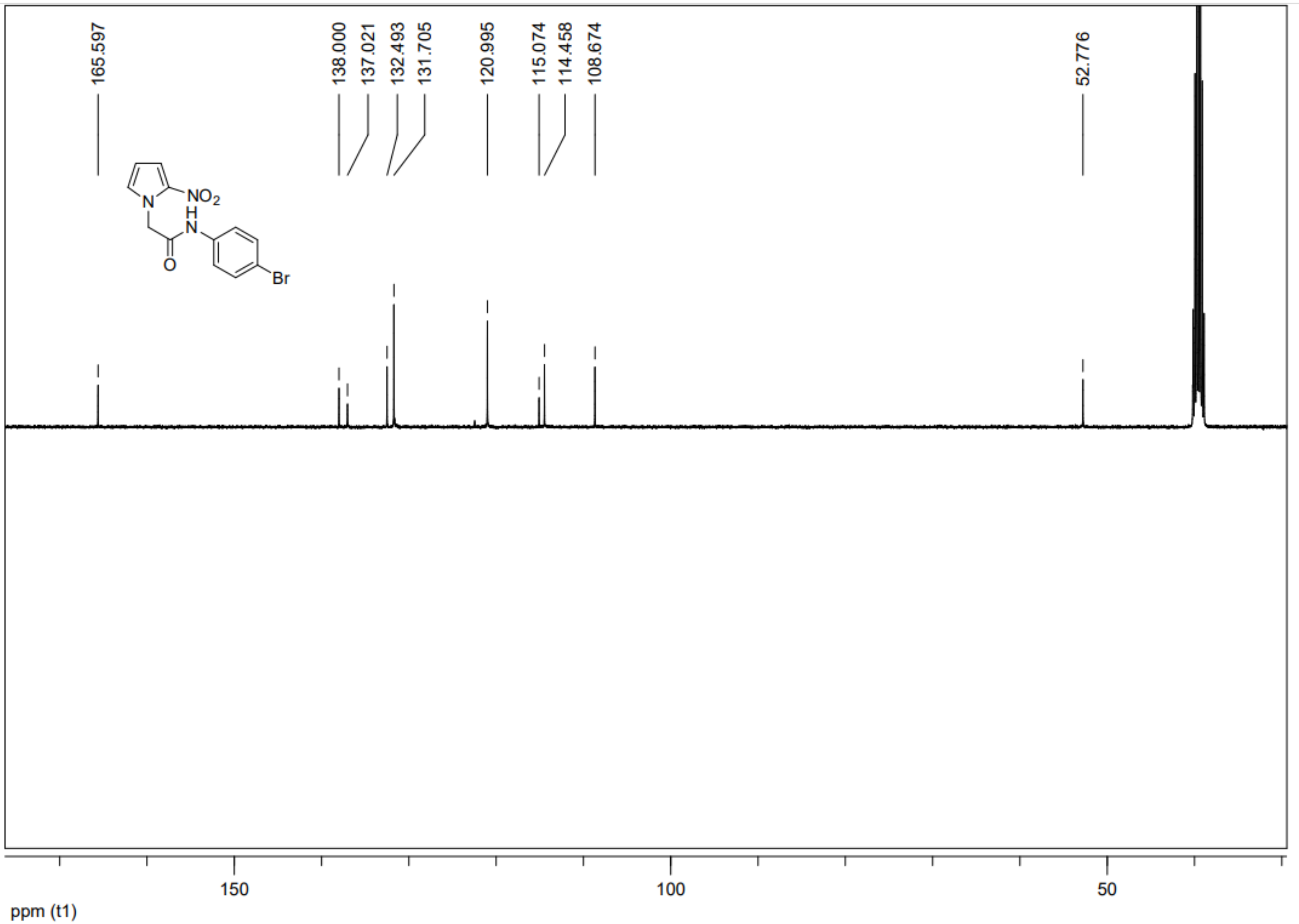

***N*-(2-Chlorophenyl)-2-(2-nitro-1*H*-pyrrol-1-yl)acetamide (17)**

Yield 96% (251 mg). White solid. Mp 178-179 °C. LC-MS (ESI+) Tr 4.44 min, m/z $[M + H]^+$ 280.02. MW: 279.04 g.mol$^{-1}$. HRMS: m/z $[M + H]^+$ calcd for $[C_{12}H_{10}ClN_3O_3]^+$ : 280.0483 ; found: 280.0483.

$^{1}H$ NMR (400 MHz, DMSO-d6) δ 9.98 (s, 1H, NH), 7.68 (dd, $^{3}J_{H-H}$ = 8.1 Hz, $^{4}J_{H-H}$ = 1.4 Hz, 1H, CH), 7.51 (dd, $^{3}J_{H-H}$ = 8.1 Hz, $^{4}J_{H-H}$ = 1.2 Hz, 1H, CH), 7.39-7.37 (m, 1H, CH), 7.34-7.31 (m, 1H, CH), 7.28 (dd, $^{3}J_{H-H}$ = 4.4 Hz, $^{4}J_{H-H}$ = 2.0 Hz, 1H, CH), 7.22-7.18 (m, 1H, CH), 6.31 (dd, $^{3}J_{H-H}$ = 4.4 Hz, $^{4}J_{H-H}$ = 2.7 Hz, 1H, CH), 5.33 (s, 2H, $CH_2$).

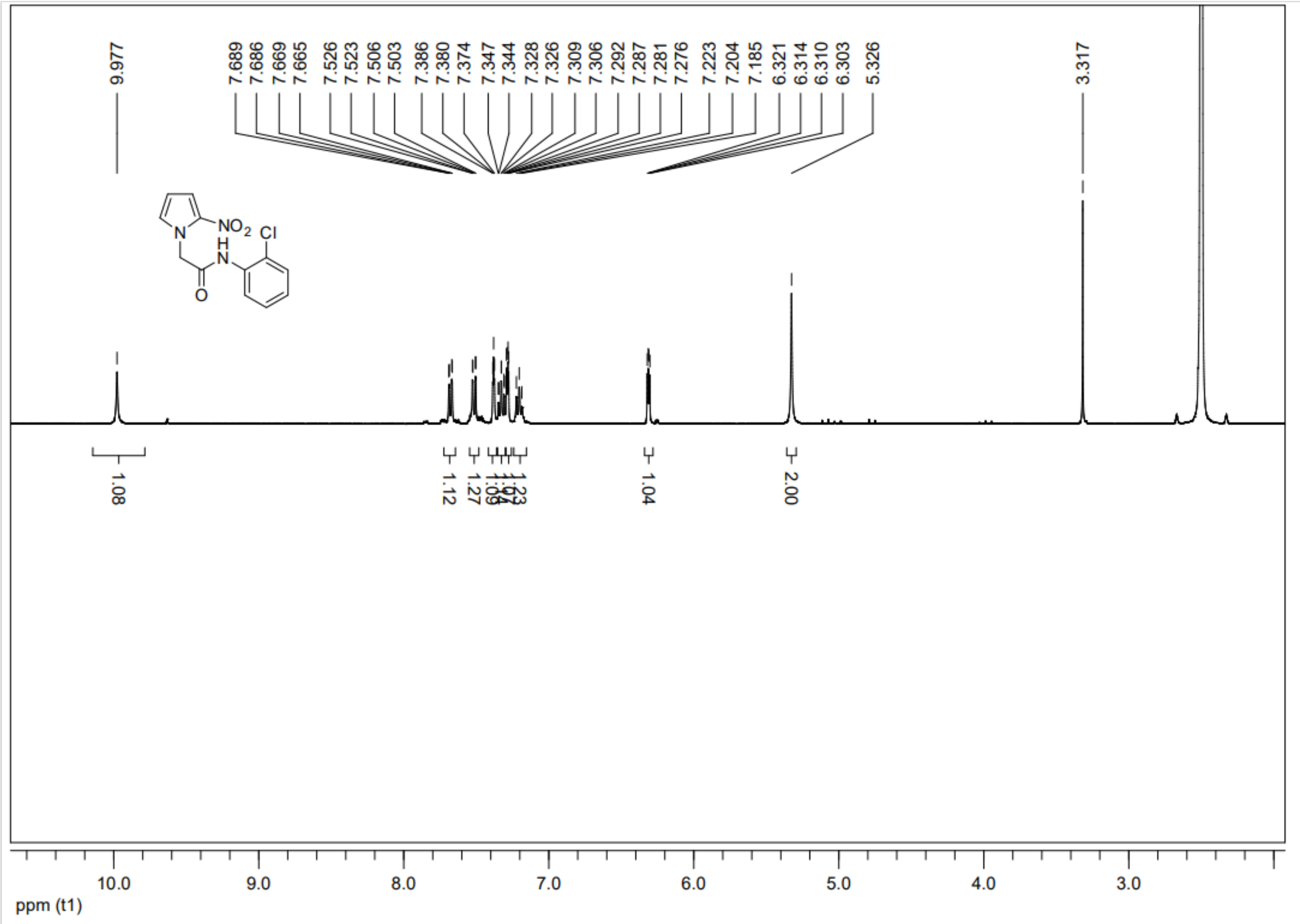

$^{13}C$ NMR (100 MHz, DMSO-d6) δ 166.1 (C), 137.0 (C), 134.4 (C), 132.4 (CH), 129.6 (CH), 127.5 (CH), 126.5 (CH), 126.2 (C), 125.9 (CH), 114.4 (CH), 108.6 (CH), 52.6 ($CH_2$).

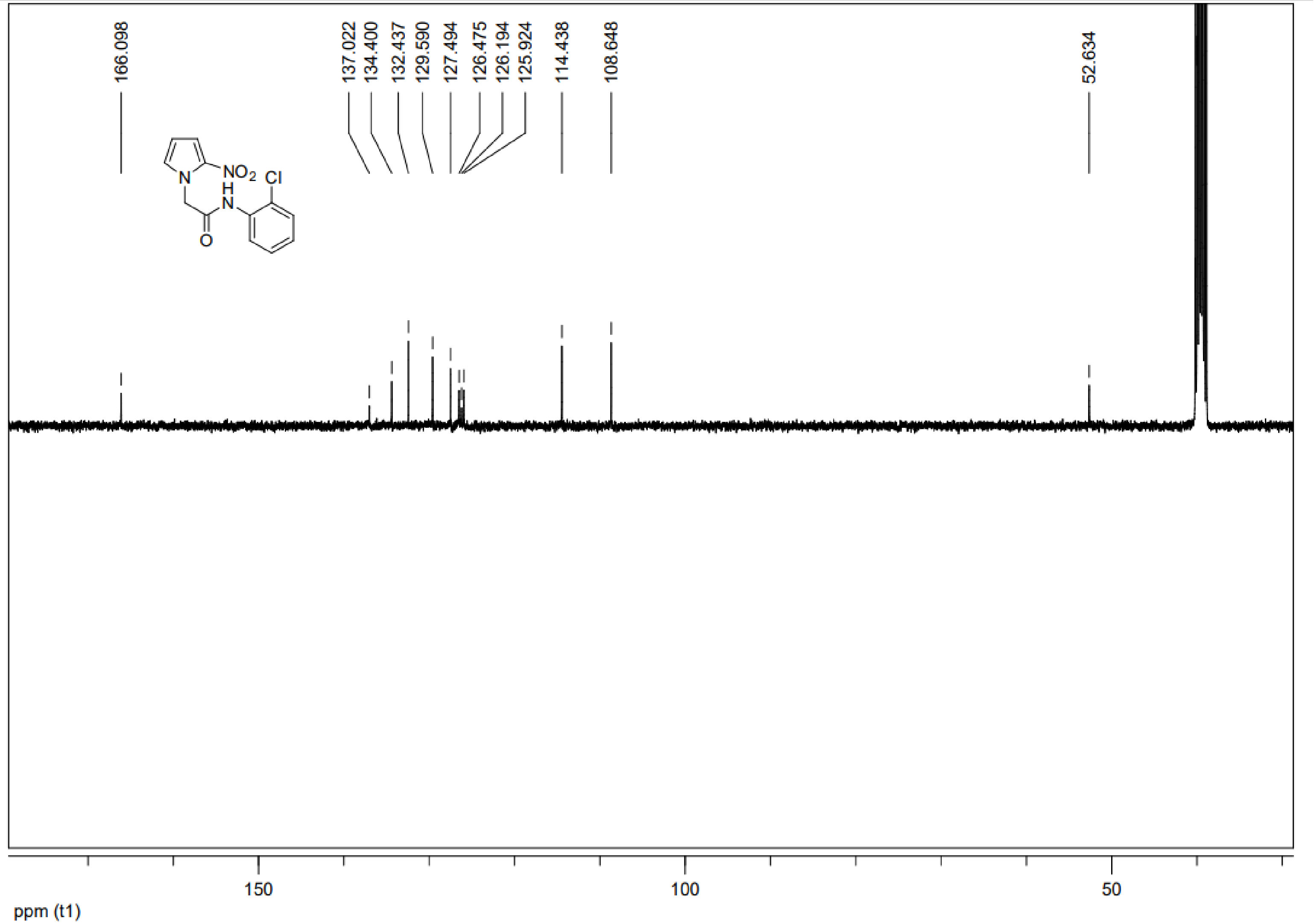

**2-(2-Nitro-1*H*-pyrrol-1-yl)-*N*-[3-(trifluoromethyl)phenyl]acetamide (18)**

Yield 81% (217 mg). White solid. Mp 202-203 °C.

LC-MS (ESI+) Tr 5.21 min, m/z $[M + H]^+$ 314.11. MW: 313.07 g.mol$^{-1}$. HRMS: m/z $[M + H]^+$ calcd for $[C_{13}H_{10}F_3N_3O_3]^+$ : 314.0747 ; found: 314.0745.

$^{1}$H NMR (400 MHz, DMSO-d6) δ 10.7 (s, 1H, NH), 8.06 (s, 1H, CH), 7.75-7.73 (m, 1H, CH), 7.55-7.59 (m, 1H, CH), 7.44-7.42 (m, 1H, CH), 7.37-7.35 (m, 1H, CH), 7.30 (dd, $^{3}J_{H\text{-}H}$ = 4.3 Hz, $^{4}J_{H\text{-}H}$ = 2.1 Hz, 1H, CH), 6.33 (dd, $^{3}J_{H\text{-}H}$ = 4.4 Hz, $^{4}J_{H\text{-}H}$ = 2.7 Hz, 1H, CH), 5.27 (s, 2H, $CH_2$).

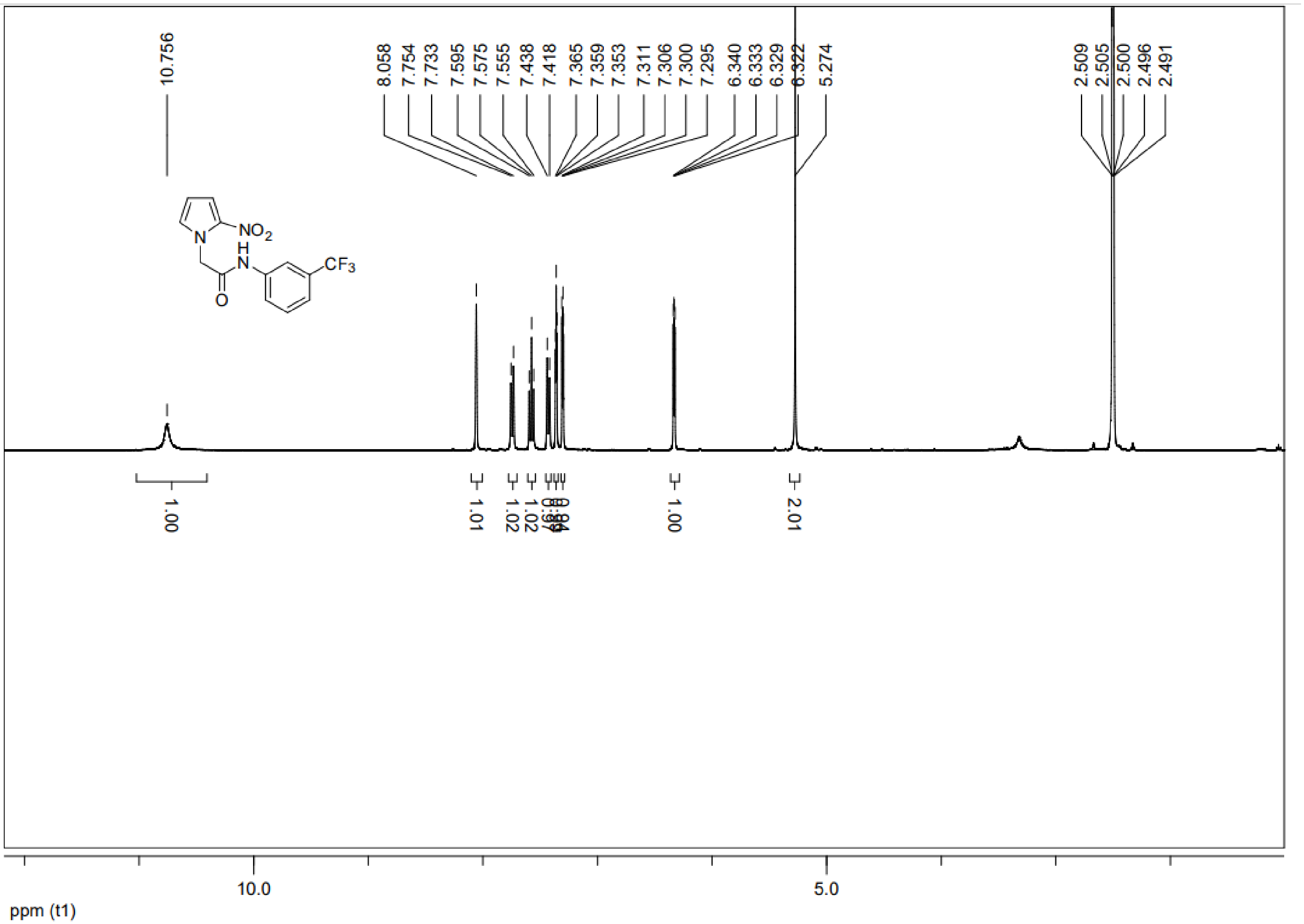

$^{13}$C NMR (100 MHz, DMSO-d6) δ 166.1 (C), 139.4 (C), 137.0 (C), 132.5 (CH), 130.2 (CH), 129.5 (q, $^{2}J_{C-F}$ = 32 Hz, C), 124.0 (q, $^{1}J_{C-F}$ = 271.0 Hz, C), 122.6 (CH), 119.8 (q, $^{3}J_{C-F}$ = 4.4 Hz, CH), 115.0 (q, $^{3}J_{C-F}$ = 3.6 Hz, CH), 114.4 (CH), 108.7 (CH), 52.8 ($CH_2$).

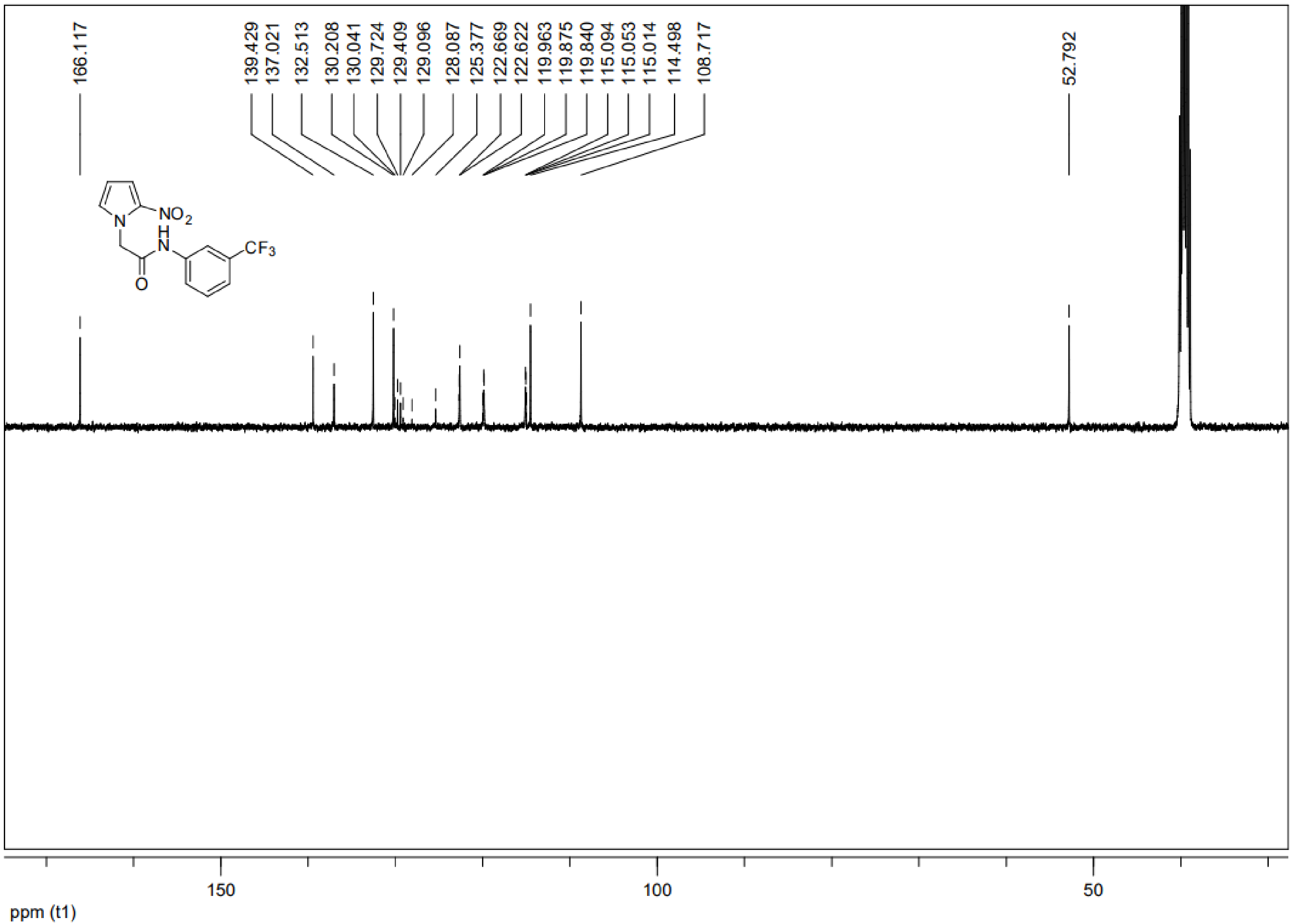

***N*-(3-Methoxyphenyl)-2-(2-nitro-1*H*-pyrrol-1-yl)acetamide (19)**

Yield 88% (217 mg). White solid. Mp 192-193 °C.

LC-MS (ESI+) Tr 4.32 min, m/z $[M + H]^+$ 276.11. MW: 275.09 $g.mol^{-1}$. HRMS: m/z $[M + H]^+$ calcd for $[C_{13}H_{13}N_3O_4]^+$ : 276.0979 ; found: 276.0979.

$^{1}H$ NMR (400 MHz, DMSO-d6) δ 10.39 (s, 1H, NH), 7.35-7.21 (m, 4H, 4CH), 7.08 (s, 1H, CH), 6.64 (s, 1H, CH), 6.32 (s, 1H, CH), 5.23 (s, 2H, $CH_2$), 3.72 (s, 3H, $CH_3$).

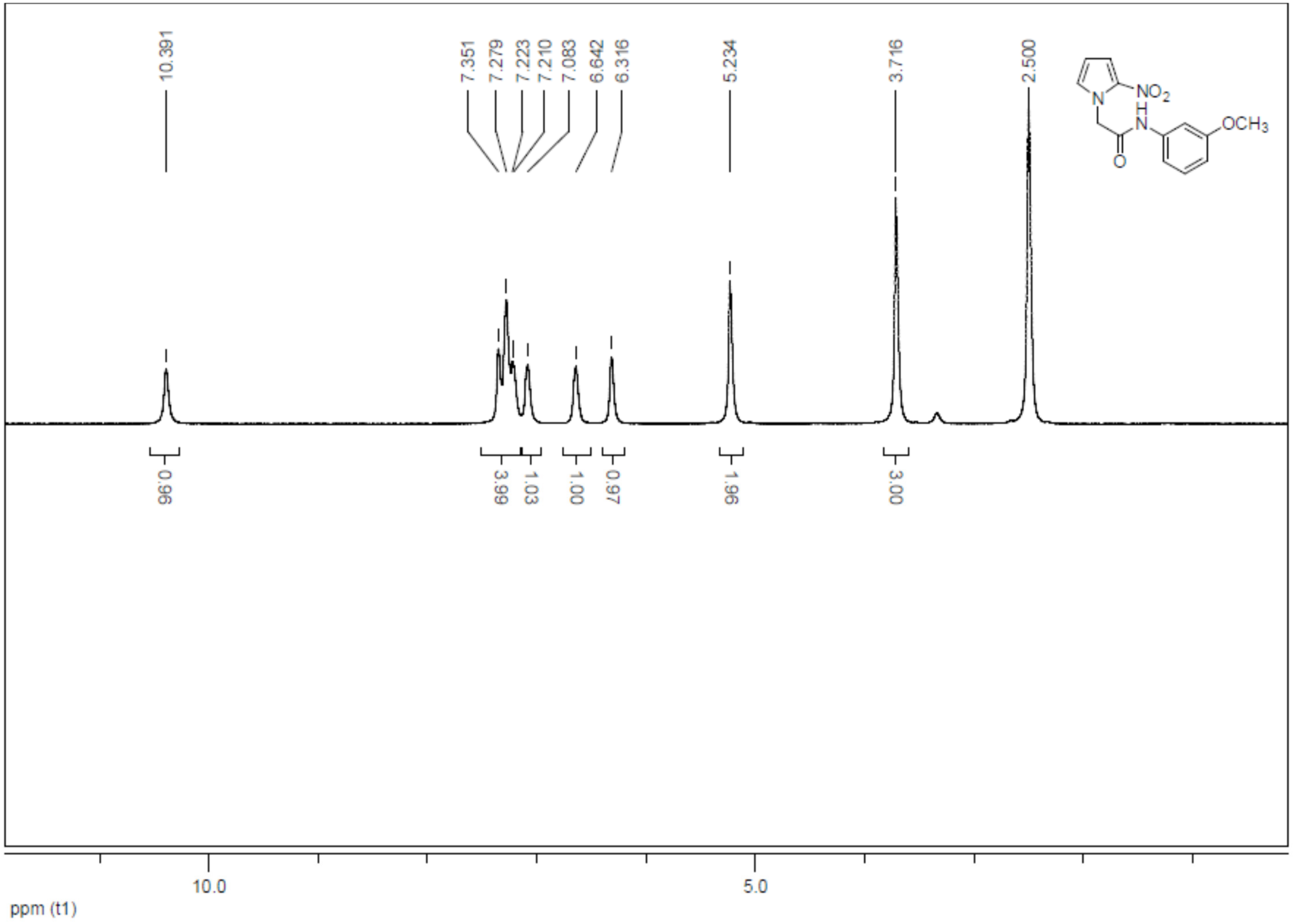

$^{13}C$ NMR (100 MHz, DMSO-d6) δ 165.4 (C), 159.6 (C), 139.8 (C), 137.0 (C), 132.5 (CH), 129.7 (CH), 114.4 (CH), 111.2 (CH), 109.0 (CH), 108.6 (CH), 104.7 (CH), 54.9 ($CH_3$), 52.8 ($CH_2$).

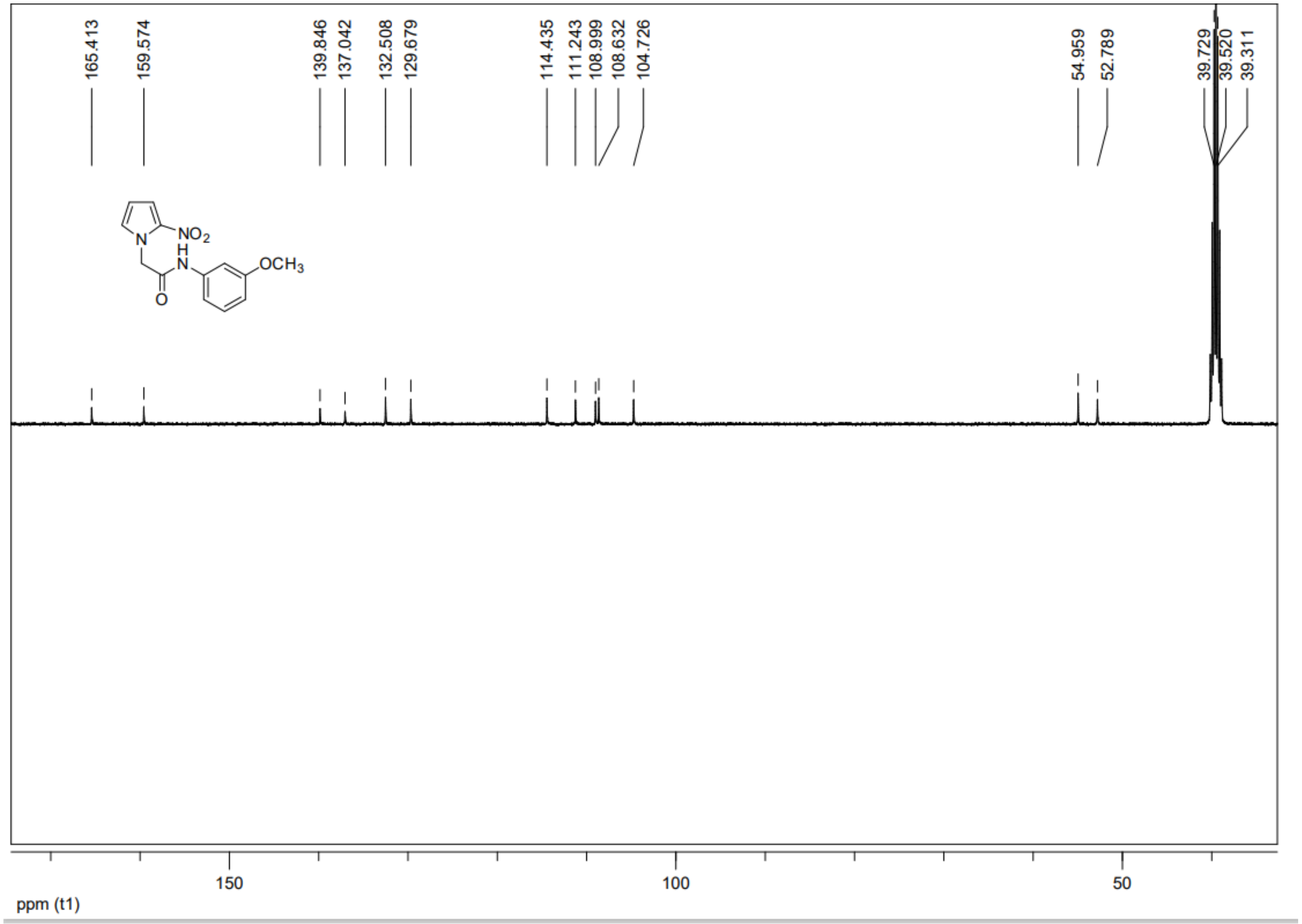

***N*-Isopropyl-2-(2-nitro-1*H*-pyrrol-1-yl)acetamide (20)**

Yield 30% (57 mg). White solid. Mp 190-191 °C. LC-MS (ESI+) Tr 3.16 min, m/z $[M + H]^+$ 212.34. MW: 211.10 g.mol$^{-1}$. HRMS: m/z $[M + H]^+$ calcd for $[C_9H_{13}N_3O_3]^+$ : 212.1030 ; found: 212.1029.

$^{1}H$ NMR (400 MHz, DMSO-d6) δ 8.09 (d, $^{3}J_{H\text{-}H}$ = 7.5 Hz, 1H, NH), 7.27 (t, $^{3}J_{H\text{-}H}$ = 2.4 Hz, 1H, CH), 7.22 (dd, $^{3}J_{H\text{-}H}$ = 4.2 Hz, $^{4}J_{H\text{-}H}$ = 2.1 Hz, 1H, CH), 6.25 (dd, $^{3}J_{H\text{-}H}$ = 4.2 Hz, $^{4}J_{H\text{-}H}$ = 2.7 Hz, 1H, CH), 4.95 (s, 2H, $CH_2$), 3.86-3.78 (m, 1H, CH), 1.07 (d, $^{3}J_{H\text{-}H}$ = 6.6 Hz, 6H, $2CH_3$).

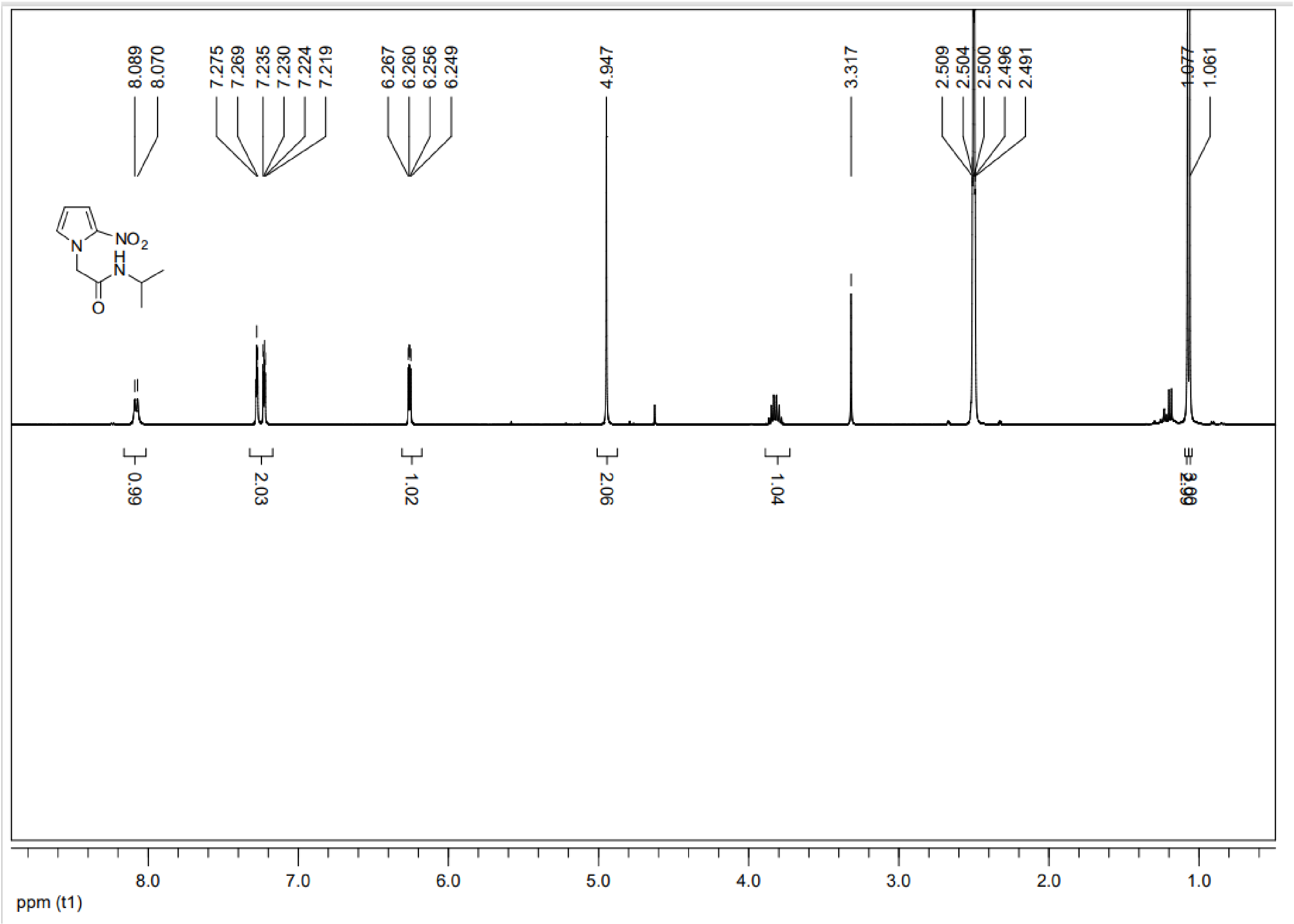

$^{13}C$ NMR (100 MHz, DMSO-d6) δ 165.3 (C), 137.1 (C), 132.3 (CH), 114.2 (CH), 108.3 (CH), 52.1 (CH), 40.7 ($CH_2$), 22.4 ($2CH_3$).

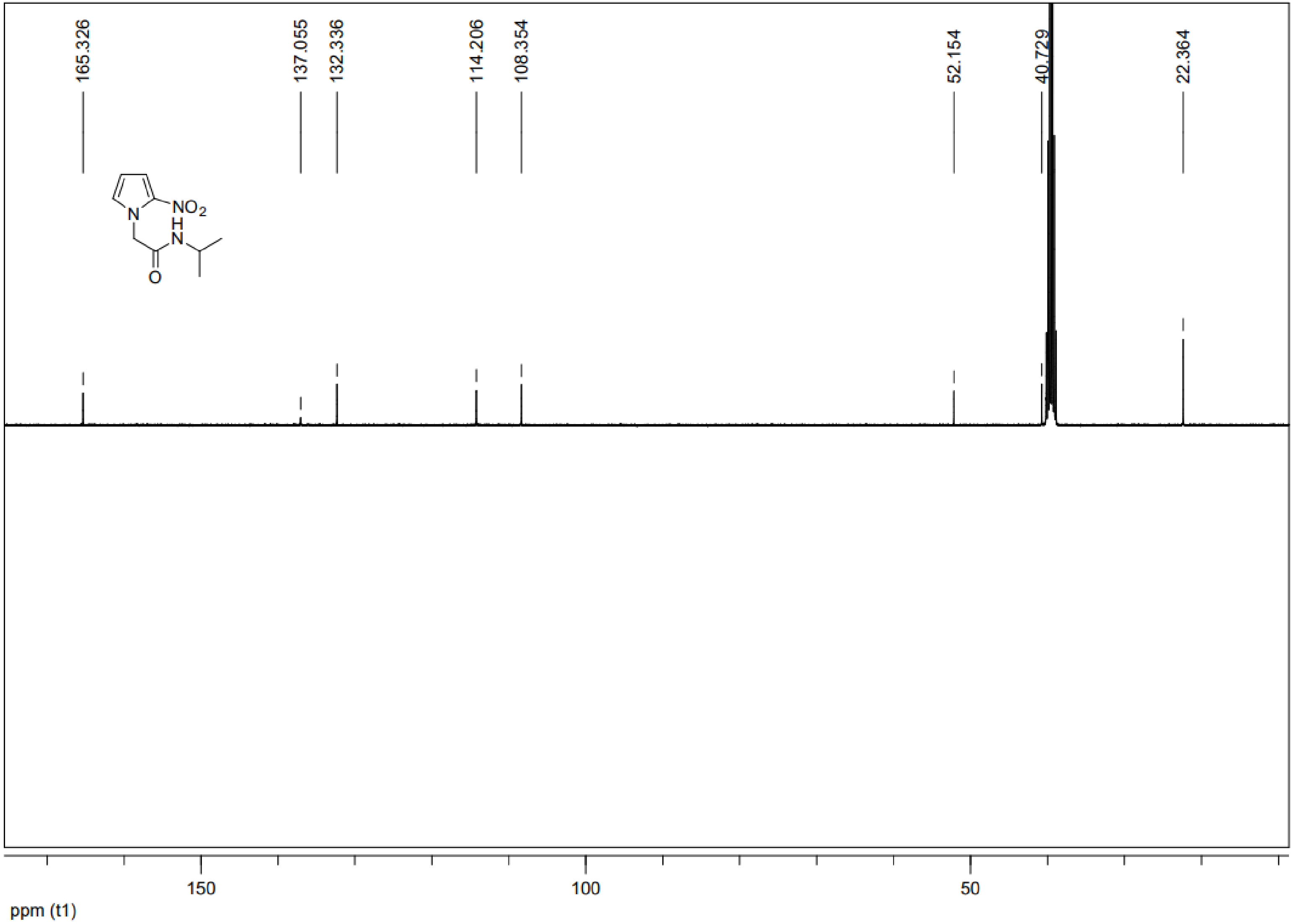

**N-Cyclopentyl-2-(2-nitro-1H-pyrrol-1-yl)acetamide (21)**

Yield 59% (125 mg). White solid. Mp 190-191 °C.

LC-MS (ESI+) Tr 3.85 min, m/z $[M + H]^+$ 238.24. MW: 237.11 g.mol$^{-1}$. HRMS: m/z $[M + H]^+$ calcd for $[C_{11}H_{15}N_3O_3]^+$ : 238.1186 ; found: 238.1191.

$^1H$ NMR (400 MHz, DMSO-d6) δ 8.17 (d, $^3J_{H-H}$ = 7.3 Hz, 1H, NH), 7.28 (t, $^3J_{H-H}$ = 2.2 Hz, 1H, CH), 7.23 (dd, $^3J_{H-H}$ = 4.2 Hz, $^4J_{H-H}$ = 2.1 Hz, 1H, CH), 6.26 (dd, $^3J_{H-H}$ = 4.4 Hz, $^4J_{H-H}$ = 2.7 Hz, 1H, CH), 4.96 (s, 2H, $CH_2$), 4.01-3.96 (m, 1H, CH), 1.82-1.76 (m, 2H, $CH_2$), 1.67-1.64 (m, 2H, $CH_2$), 1.53-1.49 (m, 2H, $CH_2$), 1.44-1.37 (m, 2H, $CH_2$).

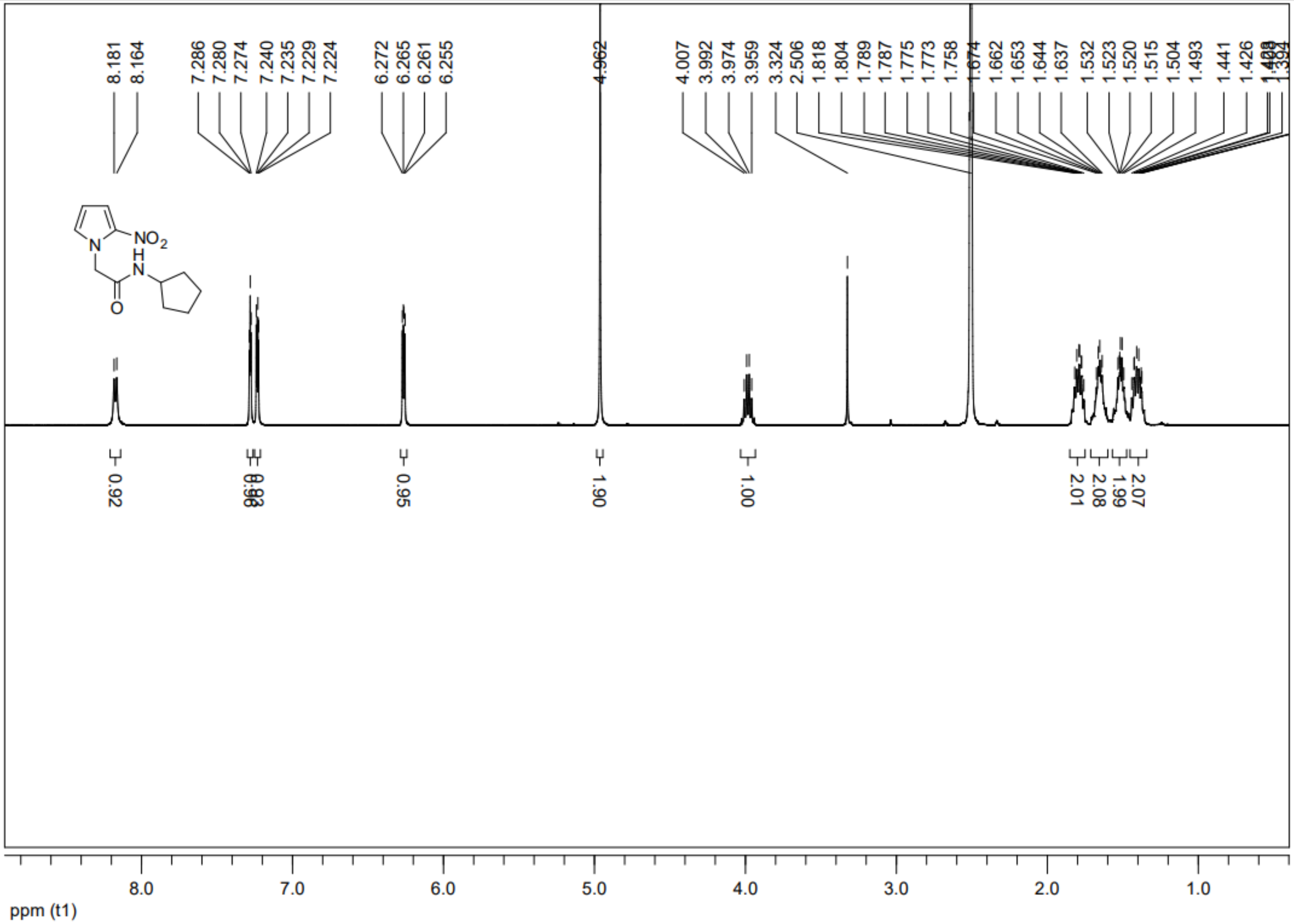

$^{13}C$ NMR (100 MHz, DMSO-d6) δ 165.7 (C), 137.1 (C), 132.3 (CH), 114.2 (CH), 108.3 (CH), 52.1 (CH), 50.5 ($CH_2$), 32.3 ($2CH_2$), 23.4 ($2CH_2$).

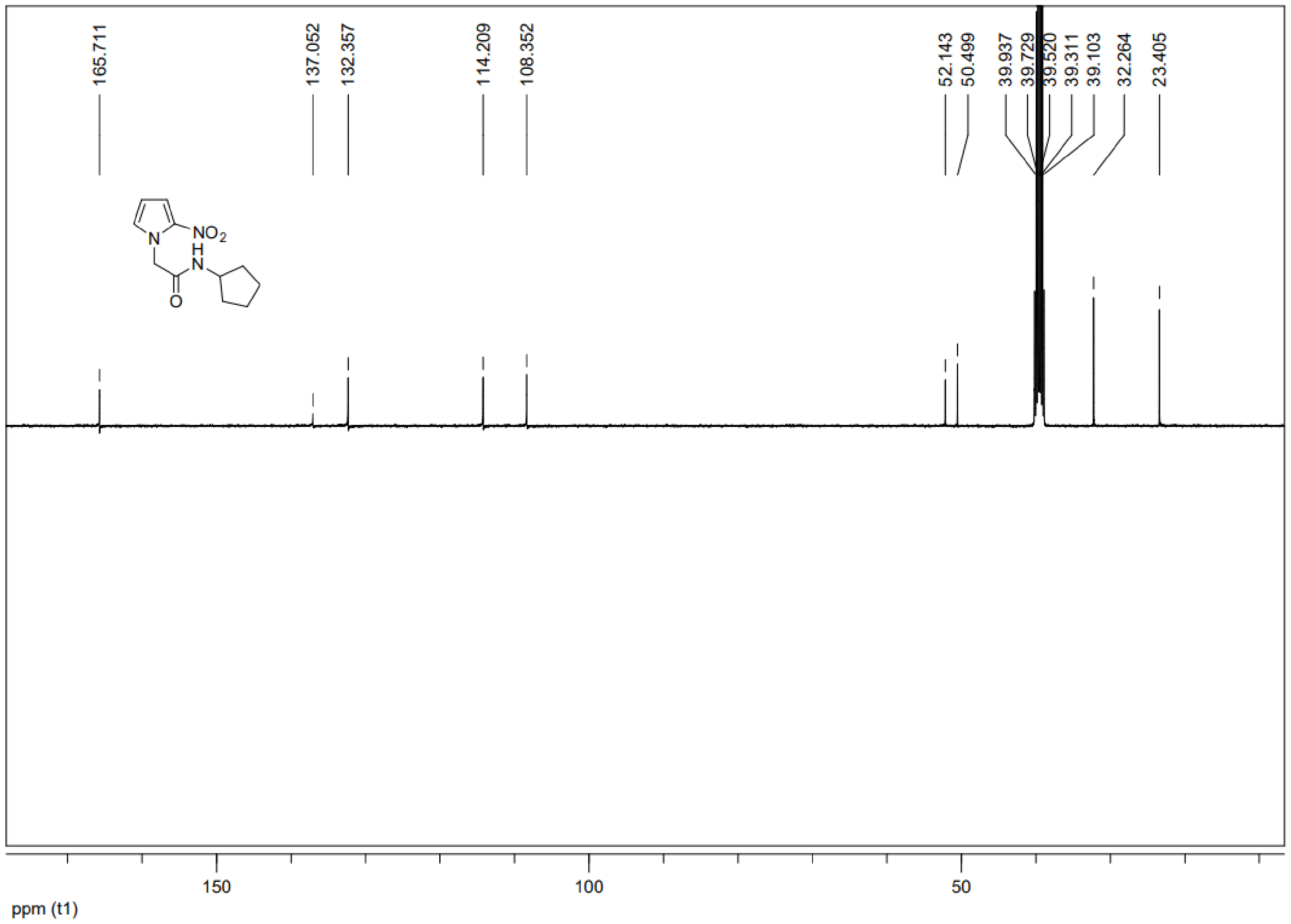

**_N_-(2-Bromo-5-fluorophenyl)-2-(2-nitro-1_H_-pyrrol-1-yl)acetamide (22)**

Yield 77% (123 mg). Yellow solid. Mp 185-186 °C.

LC-MS (ESI+) Tr 4.84 min, m/z $[M + H]^+$ 342.0. MW: 340.98 g.mol$^{-1}$. HRMS: m/z $[M + H]^+$ calcd for $[C_{12}H_9BrFN_3O_3]^+$ : 341.9884; found: 341.9877.

$^1$H NMR (400 MHz, DMSO-d6) δ 9.98 (s, 1H, NH), 7.75-7.71 (m, 1H, CH), 7.56-7.52 (m, 1H, CH), 7.07-7.02 (m, 1H, CH), 7.39 (t, $^3J_{H-H}$ = 2.3 Hz, 1H, CH), 7.30 (dd, $^3J_{H-H}$ = 4.4 Hz, $^4J_{H-H}$ = 2.0 Hz, 1H, CH), 6.33 (dd, $^3J_{H-H}$ = 4.4 Hz, $^4J_{H-H}$ = 2.7 Hz, 1H, CH), 5.35 (s, 2H, $CH_2$).

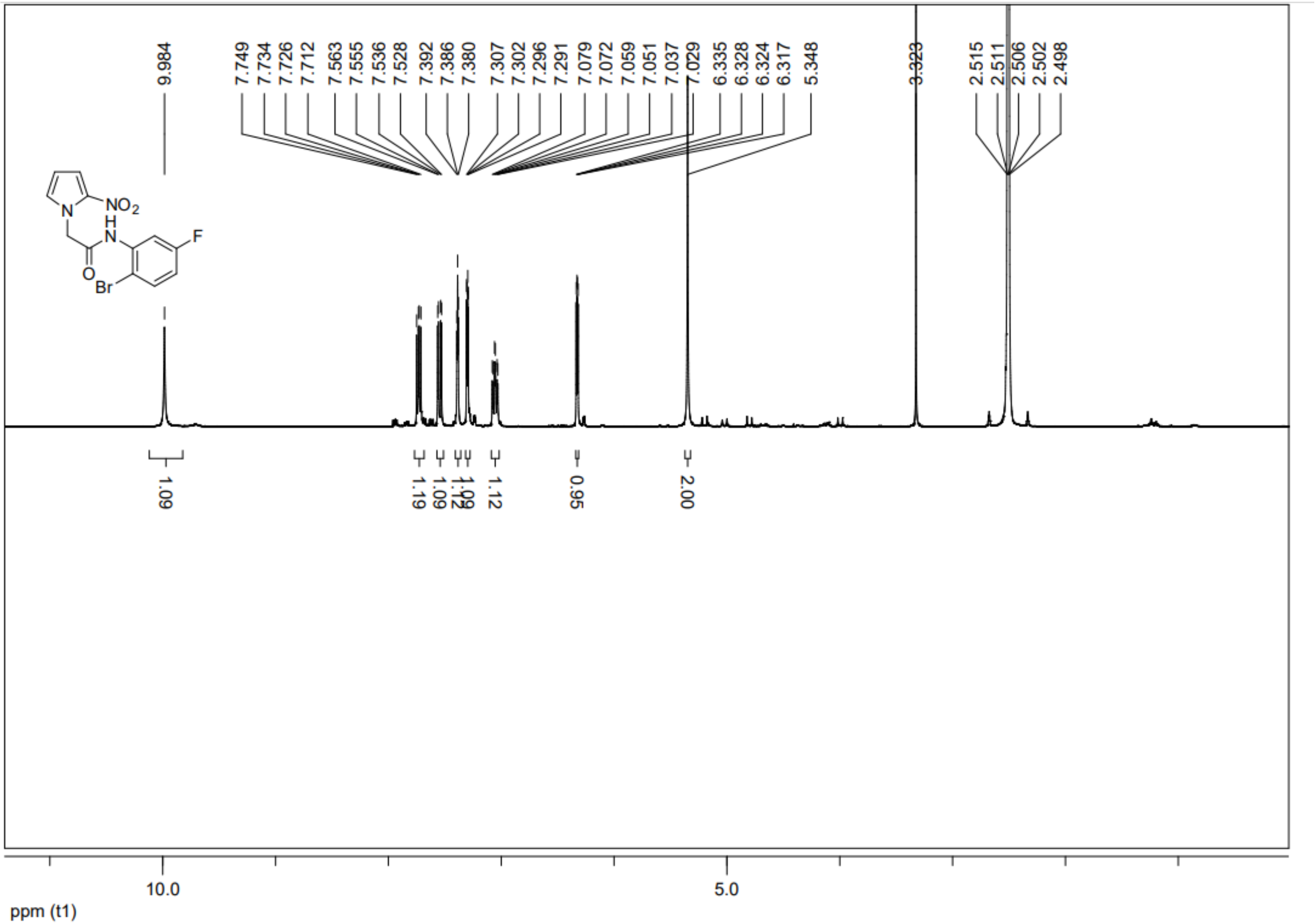

$^{13}C$ NMR (100 MHz, DMSO-d6) δ 166.4 (C), 161.0 (d, $^{1}J_{C\text{-}F}$ = 241.2 Hz, C), 137.2 (C), 137.0 (d, $^{3}J_{C\text{-}F}$ = 9.4 Hz, C), 134.0 (d, $^{3}J_{C\text{-}F}$ = 9.4 Hz, CH), 132.4 (CH), 114.5 (CH), 113.8 (d, $^{2}J_{C\text{-}F}$ = 22.5 Hz, CH), 112.6 (d, $^{2}J_{C\text{-}F}$ = 26.1 Hz, CH), 110.9 (C), 108.7 (CH), 52.7 ($CH_2$).

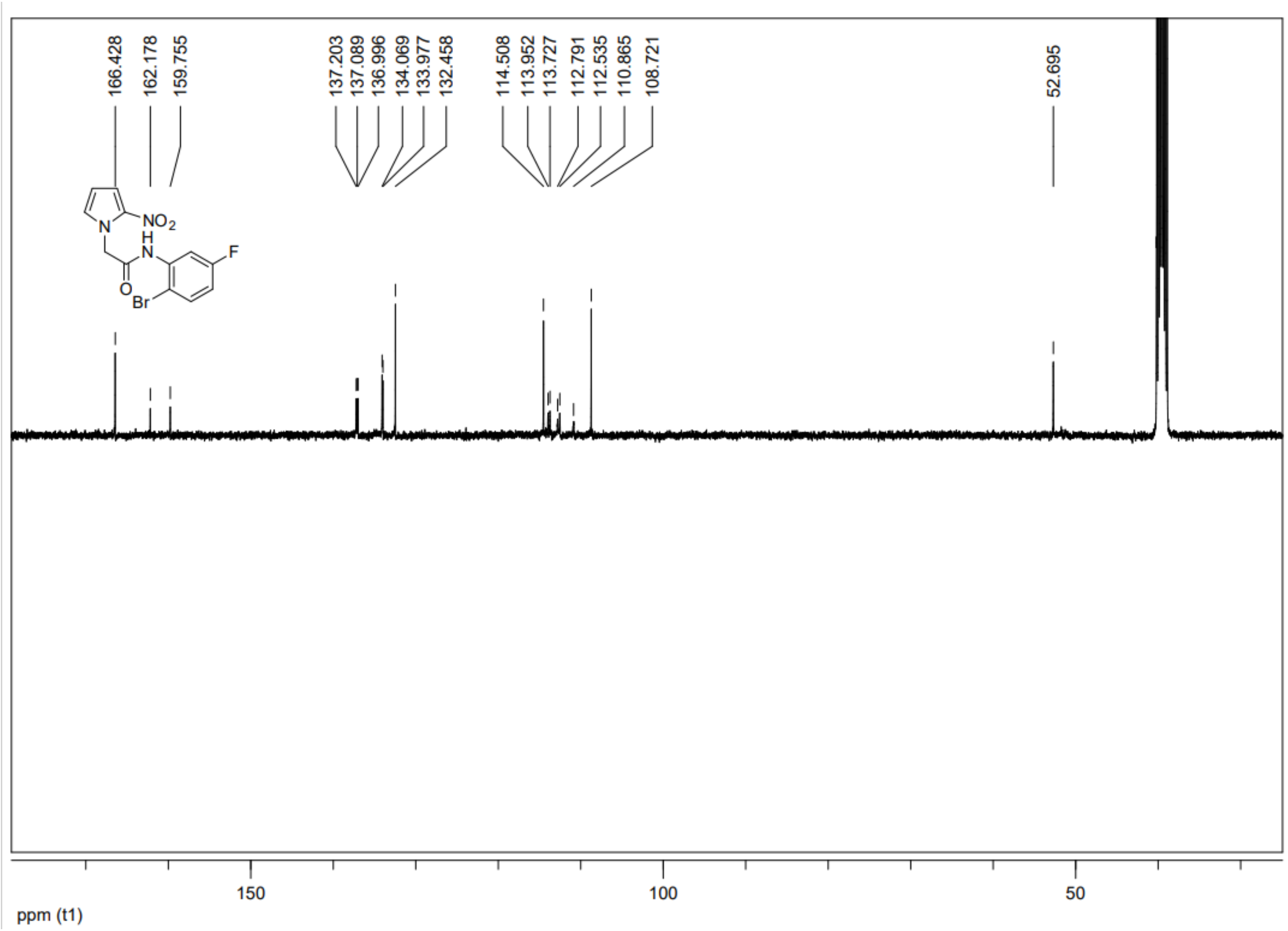

Dose response curves for compound **3**, **4**, **15**, **16**, **17**, **19** against T. cruzi X10/7 intracellular amastigotes. Left panels show percent inhibition of intracellular amastigotes (normalised to DMSO (0%) and nifurtimox controls (100%)). Right hand panels show inhibition of Vero host cell growth.

Compound **3**

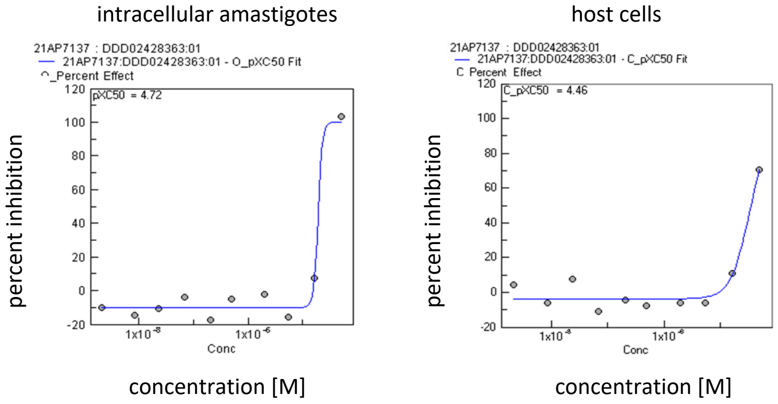


Compound **4**

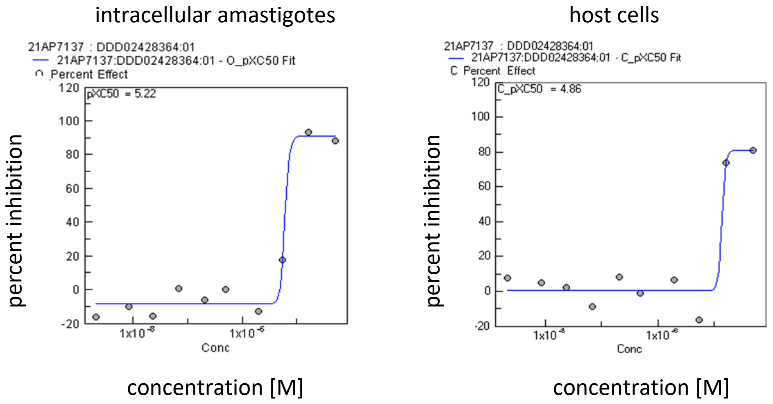


Compound **15**

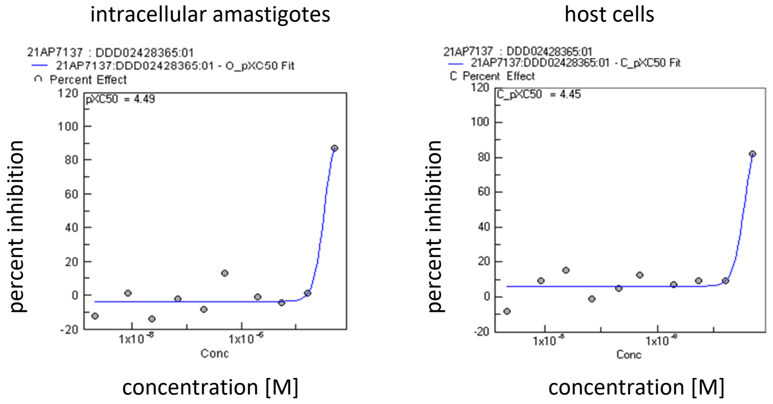

## Compound **16**

intracellular amastigotes | host cells

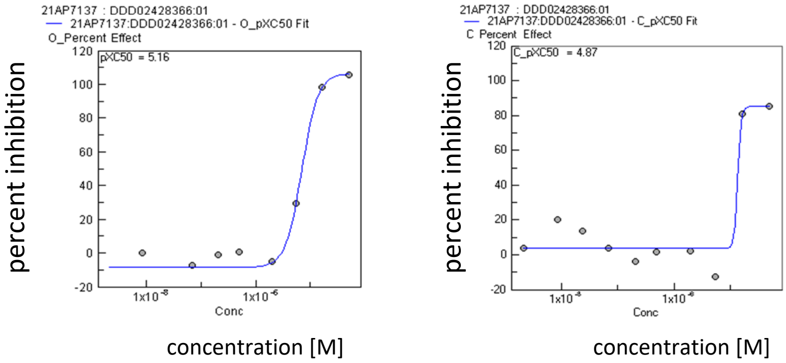


## Compound **17**

intracellular amastigotes | host cells

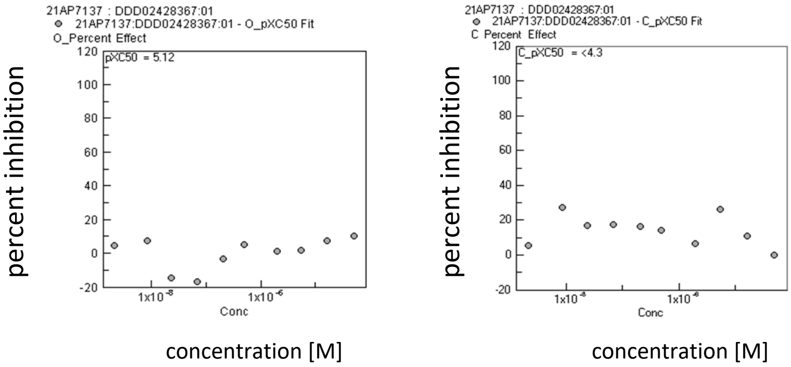


## Compound **19**

intracellular amastigotes | host cells

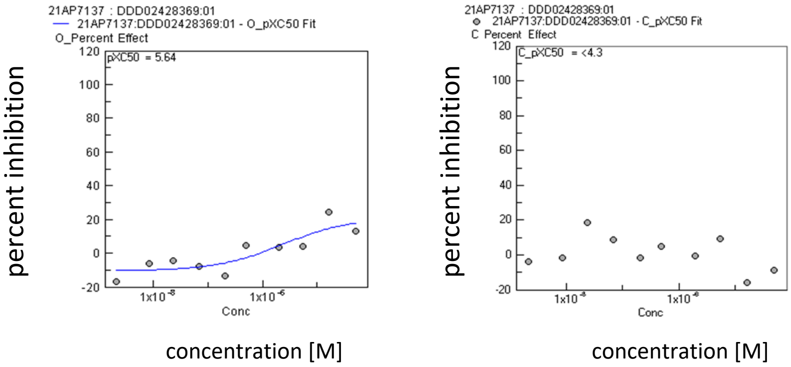

Supplement: Supplementary file 1 [file molecules-27-02163-s001.zip › molecules-1634600-supplementary.pdf]
